# Functional Connectivity of EEG Signals Under Laser Stimulation in Migraine

**DOI:** 10.3389/fnhum.2015.00640

**Published:** 2015-11-24

**Authors:** Marina de Tommaso, Gabriele Trotta, Eleonora Vecchio, Katia Ricci, Frederik Van de Steen, Anna Montemurno, Marta Lorenzo, Daniele Marinazzo, Roberto Bellotti, Sebastiano Stramaglia

**Affiliations:** ^1^Basic Medical Neuroscience and Sensory System Department, Bari Aldo Moro University, Bari, Italy; ^2^TIRES Center, Bari Aldo Moro University, Bari, Italy; ^3^Physics Department, Istituto Nazionale Di Fisica Nucleare Sezione di Bari, Bari Aldo Moro University, Bari, Italy; ^4^BCAM Basque Center for Applied Mathematics, Bilbao, Spain; ^5^Data Analysis Department, Faculty of Psychological and Pedagogical Sciences 1, Ghent University, Ghent, Belgium

**Keywords:** migraine, laser-evoked potentials, synchronization, Granger causality, habituation

## Abstract

In previous studies, migraine patients showed abnormalities in pain-related evoked responses, as reduced habituation to repetitive stimulation. In this study, we aimed to apply a novel analysis of EEG bands synchronization and directed dynamical influences under painful stimuli in migraine patients compared to non-migraine healthy volunteers. Thirty-one migraine without aura outpatients (MIGR) were evaluated and compared to 19 controls (CONT). The right hand was stimulated by means of 30 consecutive CO_2_ laser stimuli. EEG signal was examined by means of Morlet wavelet, synchronization entropy (SE), and Granger causality (GC), and the statistically validated results were mapped on the corresponding scalp locations. The vertex complex of averaged laser-evoked responses (LEPs) showed reduced habituation compared to CONT. In the prestimulus phase, enhanced SE in the 0, 5–30 Hz range was present in MIGR and CONT between the bilateral temporal–parietal and the frontal regions around the midline. Migraine patients showed an anticipation of EEG changes preceding the painful stimulation compared to CONT. In the poststimulus phase, the same cortical areas were more connected in MIGR vs CONT. In both groups of patients and CONT, the habituation index was negatively correlated with the GC scores. A different pattern of cortical activation after painful stimulation was present in migraine. The increase in cortical connections during repetitive painful stimulation may subtend the phenomenon of LEPs reduced habituation. Brain network analysis may give an aid in understanding subtle changes of pain processing under laser stimuli in migraine patients.

## Introduction

Migraine is a chronic disorder of neurovascular origin, characterized by abnormal neuronal excitability and altered processing of multimodal stimuli (de Tommaso et al., 2014). Pain modulation is different in migraine patients in respect to controls (CONT), and this may favor central sensitization and evolution into chronic form (Louter et al., 2013). Neurophysiological studies employing laser-evoked potentials, which consist of cortical responses under stimuli selective for nociceptive afferents (Treede et al., 2003), showed that central processing of painful stimuli is different in migraine compared to non-migraine subjects, following an attentive attraction scarcely reduced by habituation across repetitive stimulation or distraction induced by contemporary cognitive tasks (de Tommaso, 2008). The mechanisms underlying noxious stimuli processing are interesting in view of the involvement of cortical areas potentially responsible for pain modulation and central sensitization phenomena augmentation (Garcia-Larrea et al., 2003). Previous studies based on the analysis of event-related modulation of EEG activity using time–frequency analysis by a complex Morlet wavelet followed by a measure of predictability showed that the organization of the local activity in cortex seen in CONT after the painful stimulation was less evident in migraine, as a sign of an inadequate cortical reactivity to pain (de Tommaso et al., 2005a). Methods able to detect subtle changes of EEG rhythms under painful stimulation may improve the knowledge of mechanisms of pain processing in normal subjects and patients with chronic pain syndromes. The study of the dynamical relationships between signals recorded at different scalp locations can help to confirm and formulate hypotheses on the physiological mechanisms related to stimuli processing. Correlations, spectral coherence and phase synchronization, which allow to understand the extent to which two variables are statistically connected or shared influence by a third variable, together with analyses of the directionality of these dynamical interactions (Granger, 1969; Blinowska et al., 2004; Dhamala et al., 2008; Marinazzo et al., 2008a; Friston, 2011), may potentially contribute to the understanding and the mechanism of pain processing in migraine. Functional connectivity and information transfer within local networks in regions associated with memory, emotions and motivation were reported to be closely involved in pain processing and modulation determining the efficiency of analgesia (Hashmi et al., 2014). Moreover, attention modulation during painful stimulation changed functional connections between cortical pain-related structures, primary somatosensory, parasylvian, and medial frontal cortex, in epileptic patients examined through subdural electrodes implanted for treatment of epilepsy (Ohara et al., 2006). The complex thalamocortical dyshrhytmia causing a different sensory processing in migraine in respect to CONT involves also reactions to somatosensory nociceptive stimuli (de Tommaso et al., 2014). Studies investigating resting-state functional connectivity in functional magnetic resonance imaging (fMRI) showed atypical functional connectivity of sensory processing regions in migraine patients (Schwedt et al., 2015; Tso et al., 2015). In particular the cortical areas involved in pain processing were found affected by altered functional connections, with special regard to chronic migraine and patients with evident symptoms of central sensitization (Schwedt et al., 2013, 2014; Colombo et al., 2015; Liu et al., 2015), leading to formulate hypotheses on abnormal dynamic changes within pain network in the course of multimodal and specifically noxious stimuli processing. Functional and effective connectivity in terms of synchronization and information transfer were able to reveal differences in visual reactivity between migraine patients and CONT (Angelini et al., 2004; de Tommaso et al., 2013); these methods may thus presumably outline a different way to process nociceptive laser stimuli in migraine, giving further knowledge on how the cortex changes its interconnections under painful inputs. The aim of the present study was to apply functional and effective connectivity analysis to laser-evoked responses (LEPs), in order to detect subtle differences in cortical functioning between migraine patients and healthy volunteers.

## Methods

### Subjects

Thirty-one migraine without aura outpatients (MIGR) (Headache Classification Committee, 2004) came for the first time to the Neurophysiopathology of Pain Unit (Basic Medical Science, Neuroscience and Sensory System – SMBNOS – Department of Bari University) from January 2012 to January 2013; provided with a reliable headache diary, were selected and recorded in the interictal state, at least 72 h after the last attack, and more than 48 before the next one, ascertained by direct or telephone contact. The patients were 28 women and three males, aged 37.56 ± 11.51. The mean headache duration was 13.1 years (SD 11.57), and the mean headache frequency in the last three months was 6.34 days with headache in a month (3.4 SD). No patient experienced exclusively unilateral migraine. The recording session was performed 78.2 + 2.1 h after the last attack and 49.1 + 2.8 h before the next one. Nineteen healthy volunteers were selected (CONT), on the basis of the absence of personal and first degree familiar history for migraine. They were 18 females and one male, aged 32.5 + 7.34. Exclusion criteria were analgesic, non-steroidal drugs or triptans intake in the last 72 h, CNS acting drugs and any preventive therapy for migraine in the last 3 months and history of general medical and neurological or psychiatric diseases. Three patients reported preventive treatment in the recent past, which had been discontinued at least 3 months before the first access to our center, for not compliance in two cases and scarce efficacy in the other one.

#### Laser Stimulation and Experimental Procedure

The pain stimulus consisted of laser pulses (wavelength 10.6 μm) that were generated by a CO_2_ laser (Neurolas Electronic Engineering Florence, Italy). The stimulation site was visualized using a He–Ne laser beam. After each stimulation, the laser beam was slightly shifted to a nearby site to avoid nociceptor sensitization and skin damage.

The diameter of the laser beam was 2.5 mm, and the duration of the stimulus pulse was 30 ms. To define the pain threshold, single pulses were presented in a random order at four to five different intensities at 1.5 W intervals. The subjects were requested to report the quality of sensation and the pain threshold, which was expressed as the laser intensity (expressed in Watts) that produced a pinprick sensation followed by a burning sensation. Thirty consecutive laser stimuli were then delivered at an intensity level set one step (1.5 W) above the pain threshold at an inter-stimulus interval of 10 s. We evaluated LEP habituation within single trials, in accordance with previous studies to minimize exam duration and distress (de Tommaso et al., 2015). The dorsum of the right hand was stimulated in all patients and CONT. The patients were requested to rate the laser pain intensity at the end of the stimulation series according to a 0–100 visual analogical scale in which 100 represented maximal pain, shown in intense red color.

We invited patients to pay attention to the stimuli and also gave a warning prior to the stimulus in order to avoid distraction and blinking. The study was approved by the Ethic Committee of the Bari Policlinico General Hospital, and subjects signed an informed consent for a study on the psychophysical properties of pain processing. It was conducted in accord with Helsinki declaration.

#### Recording

In addition to the 19 standard positions of the international 10–20 system, 37 additional electrodes were placed on the *x*, *y*, and *z* coordinates provided by the advanced source analysis (ASA) software (ASA Version 4.7; ANT Software, Enschede, Netherlands).^1^ The reference electrode was placed on the nose (Treede et al., 2003), the ground electrode was in Fpz, and one electrode was placed above the right eyebrow for electro-oculogram (EOG) recording. Impedance was kept at 10 kΩ or less. The EEG and EOG signals were amplified with a bandpass of 0.5–80 Hz, digitized at 250 Hz, and stored on a biopotential analyzer (Micromed System Plus; Micromed, Mogliano Veneto, Italy).^2^

### Analysis

All recordings were preliminarly analyzed by the neurologists, who considered the LEP recordings of 1 s, including 100 ms of prestimulus time, at a sampling rate of 256 Hz. All LEP recordings containing transient signals that exceeded 65 μV in any channel were excluded from the average by an automatic artifact rejection algorithm. Other artifacts were visually inspected. For each stimulation site, we evaluated the averages of at least 21 valid (artifact-free) responses, divided into blocks of at least seven artifact-free responses. The N1 component was analyzed at T3-Fz, and the N2 and P2 components were analyzed at the vertex (Cz-nasion). The absolute latencies of the scalp potentials were measured at the highest peak of each response component. The amplitude of each wave was measured from the baseline, in an automatic way by calculating the average signal on the entire sweep and subtracting this average signal from the trace. The peak-to-peak amplitude was taken into consideration for the vertex biphasic LEP component (N2P2). To assess N2P2 habituation, we decided to consider only the initial and final block of single responses, considering that in migraine the trend of LEPs amplitude changes is quite irregular in the course of the stimulation, while reduced habituation is expressed by the lack of significant amplitude modification between the first and the last responses (de Tommaso et al., 2015). We simplified the habituation pattern computing the percentual difference between the LEP amplitudes obtained in the first and third blocks of the averaged responses relative to the first block (first response − third response × 100/first response). This value was defined as the habituation index (HI). Positive values corresponded to a reduction of the N2P2 amplitudes from the first to the third stimulation series, and negative values expressed N2P2 facilitation (de Tommaso et al., 2015).

#### Wavelet Analysis

A wavelet analysis was used in order to estimate modifications over time of the EEG frequencies. The Morlet wavelet was implemented, in order to better highlight rapid changes in the composition of the EEG signal, whose functional form is reported in Datasheet S1 in Supplementary Material, and the wavelet transform was implemented in MATLAB (The MathWorks, Natick, MA, USA).^3^ A constant ratio was chosen, and the center oscillation frequencies changed in the interval 0.5–30 Hz in steps of 0.25 Hz. We also evaluated for each frequency band the line of maxima, which was the envelope of each amplitude maximum in the band under investigation. In the present study, the 1-s-wide window preceding and following the stimulation arrival was considered. For more details on the wavelet procedure, please refer to Ohara et al. (2004).

#### Functional Connection of Cortical Areas: Synchronization Entropy

To quantify the phase synchronization, the index proposed by Tass et al. (1998) was used. The artifact-free EEG signals were filtered in each band (δ band: 0.5–4 Hz; θ band: 4.25–7 Hz; α band: 7.25–12 Hz; and β band: 12.25–30 Hz) with a second order double-sided Butterworth filter. The phase synchronization index was evaluated for all pairs of electrodes for all subjects.

The analysis of phase synchronization was conducted by imposing the two weights *m* and *n* both equal to 1 (choice justified by the common nature of the signals under examination).

#### Information Flow and Effective Connectivity: Granger Causality

In this study, effective connectivity by means of Granger causality (GC) was evaluated, whose basic idea is the following: it is possible to build an autoregressive model of a time series *X* to predict its future from its past, with a certain accuracy measured by the error ϵ_*x*_. If further information from the past of another time series *Y* is added to the model, the accuracy for this new model will be given by the error ϵ_*x*__,_*_*y*_*. If ϵ_*x*__,_*_*y*_* is significantly smaller than ϵ_*x*_, then we can say that *Y* Granger causes *X* (see Datasheet S1 in Supplementary Material).

Here the non-linear generalization of GC by Kernel methods, presented in Marinazzo et al. (2008b) was used to infer the directional information flow in non-linear systems. In order to distinguish between direct and conditional influence, a multivariate approach was employed.

In this context, the analysis was conducted by means of a RBF Gaussian kernel (Marinazzo et al., 2008b), so as to deal with all degrees of non-linearity of the extracted series *X*, with a time window of 30 samples and the width of the Gaussian equal to 7, according to the leave one out criterion. We remark that the significance (corrected for multiple comparisons) is intrinsic in the methodology described in Marinazzo et al. (2008b).

### Statistical Analysis

Latencies and amplitudes of LEPs components as well as VAS and HI values were compared among groups by means of Student’s *t*-test for unpaired data. The SPSS Version 21 was employed.

The one-way ANOVA test with the Bonferroni–Holmes correction was employed to test the significance of synchronization entropy (SE) and GC values among pairs of electrodes in single groups (pre- vs poststimulus conditions). The other statistical comparisons were stimulation between groups (migraine vs CONT in the prestimulation condition and migraine vs CONT in the poststimulation condition). In this case, the Student’s *t*-test was used, with a pure Bonferroni correction equal to

$$\begin{array}{l} \left(\text{Number of frequency bands}\right)\times {\left(\text{number of channels}\right)}^{2}=\text{4}\times 61^{2}=14884 \end{array}$$

in order to account for multiple comparisons.

In order to stress as much as possible any difference among channels behavior in populations, a three-dimensional visualization key based on the vectorial representation of statistically significant percent differences of SE and GC values was employed. In this context, any plotted link is equal to the percent difference among populations multiplied by the confidence level of such comparison.

In the total of patients and CONT, the GC values (evaluated as the average, over channels, of the total outgoing GC from each node, and averaged over the bands) were correlated with HI by means of Pearson correlation test. The same test was employed to correlate GC scores with main clinical features, as migraine frequency and illness duration.

## Results

### Averaged LEPs and Pain Rating

Statistics was performed by means of Student’s *t*-test. The laser pain threshold was similar between groups, while the laser pain rating was higher in migraine vs CONT, as expressed by 0–100 VAS. [migraine 32.2 (SD 6.7), CONT 22.2 (SD 5.6), *p* < 0.008].

The LEPs’ latencies and amplitudes appeared not significantly different between groups [N1 amplitude: CONT 6.12 μV (SD 1.22), migraine 6.34 μV (SD 1.34). n.s.; N2P2 amplitude: CONT 21.21 μV (SD 12.21), migraine 23.17 μV (SD 13.24), n.s.; N1 latency: CONT 168.12 ms (SD 20.2), migraine 165.12 ms (SD 23.3), n.s; N2 latency: CONT 221.23 (SD 31.3), migraine 228.31 (SD 29.8), n.s.; P2 latency: CONT 331.32 ms (SD 23.32), migraine 334.23 ms (SD 31.3), n.s.].

Habituation index was decreased in migraine patients in respect to CONT [migraine 10.5 (SD 11.2), CONT 35.5 (SD 20.3), *p* < 0.001).

### Wavelet Analysis

**Figure 1:**
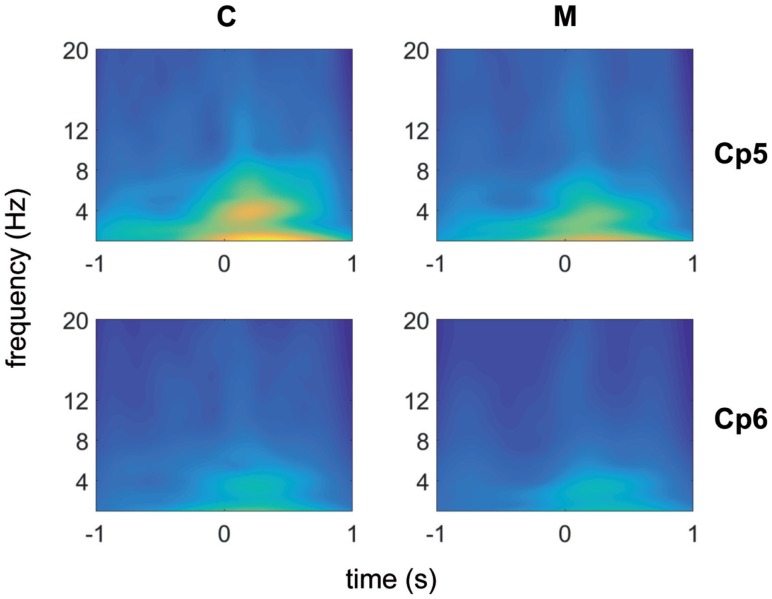
**Morlet wavelet analysis of EEG frequencies in 1 s time preceding and following laser stimuli for migraine without aura patients (no 31) (top) and control groups (no 19) (bottom)**. Average data for CP5 and CP6 channels are shown.

Using the Morlet wavelet (central frequency equal to 7, normalization factor = 1.995), each channel was analyzed by means of the wavelet transform method. The results corresponding to the channel CP6 and CP5, illustrative of almost all channels of the scalp, with the exception of those in the neighborhood of the vertex, are shown in Figure 1. We have chosen these two channels, one from the left and one from the right central–parietal areas, in which the stimulus evoked responses were more expressed.

An activity in the delta-theta band preceded the laser stimulus in both patients and CONT. A similar activity was visible in both groups in the 250–500 ms following laser stimulation. After the normalization of the wavelet images in both populations, the line of maxima for each frequency band was found for MIGR 0.8 (SD 0.03) seconds before the laser stimulus delivery, while this time in CONT was 0.3 (SD 0.03) (*p* < 0.01), so migraine patients had anticipated prestimulus EEG activation compared to CONT (Figure 1).

### Functional Connectivity Analysis: Synchronization Entropy

**Figure 2:**
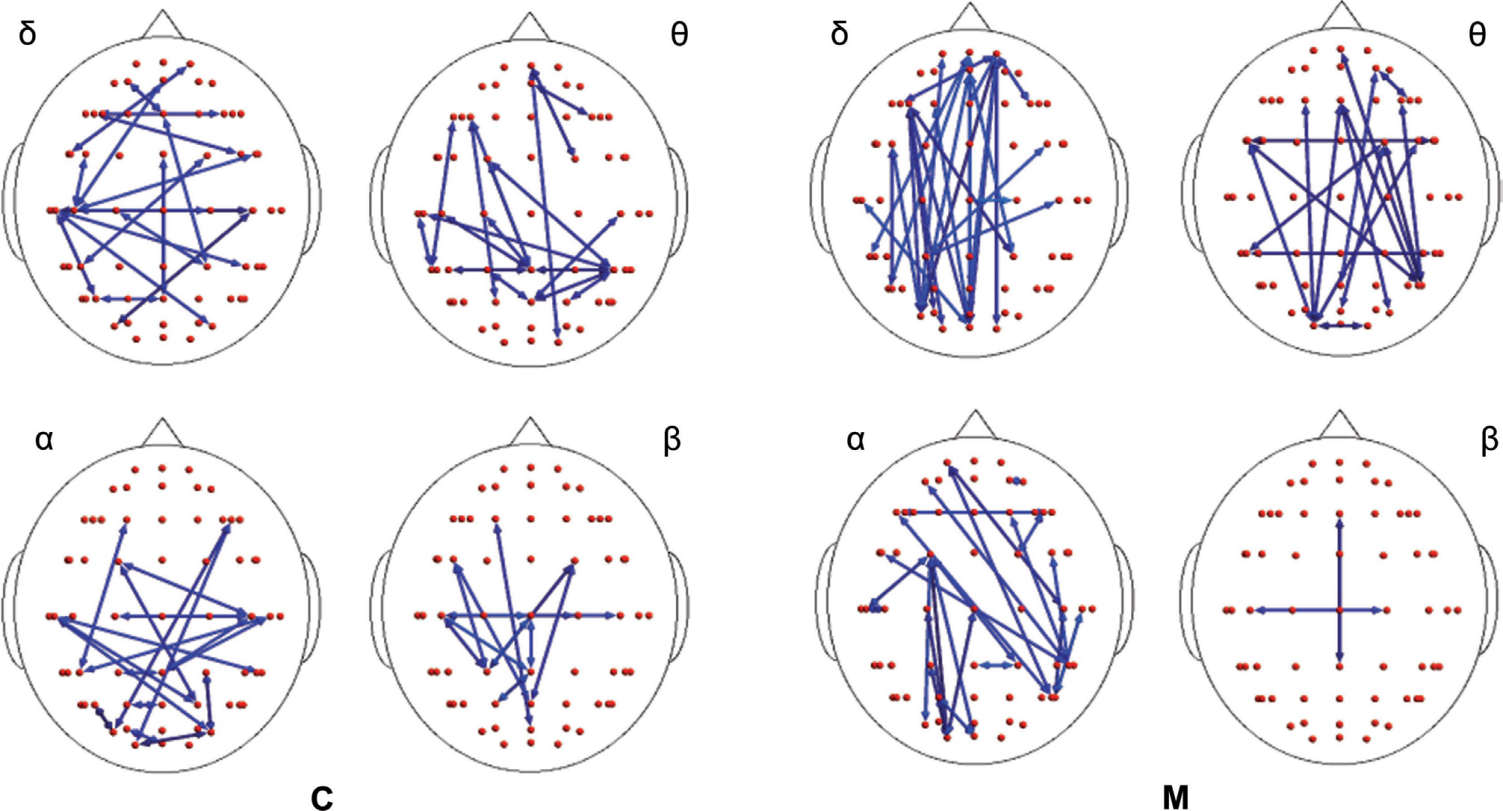
**Differences between synchronization entropy (SE) computed in the time following and that preceding the laser stimulus in control (C) and migraine (M) groups in the four bands**. The blue color expresses a statistically significant reduction of SE in the time following the painful stimuli delivering. For the details regarding the data and statistical analysis, see Tables S1–S4 in Supplementary Material.

In both migraine patients and CONT, an increased EEG synchronization of the bilateral temporal–parietal and frontal regions was present before the laser stimuli, in comparison with the poststimulus phase (Figure 2). The increase of synchronization in the prestimulus phase involved all the considered EEG bands, with the exception of beta band in CONT group, without significant differences between groups (Figure 2).

**Figure 3:**
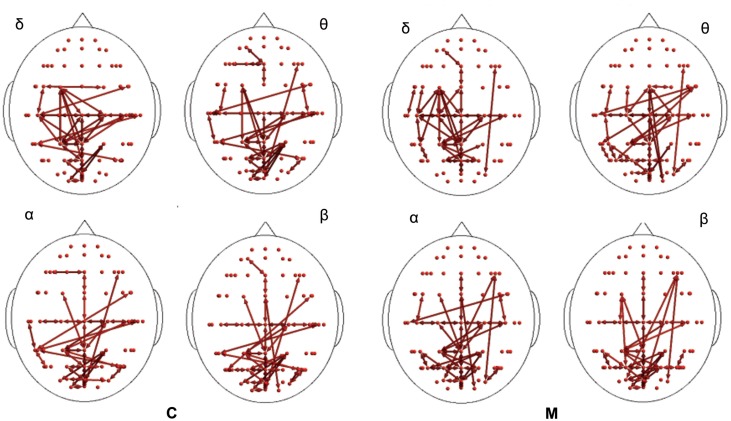
**Synchronization entropy (SE) computed 1 s after the laser stimuli**. Red arrows represent basal activity for controls (C) and migraineurs (M) in the four bands. For the details regarding the data and statistical analysis, see Tables S5 and S6 in Supplementary Material.

**Figure 4:**
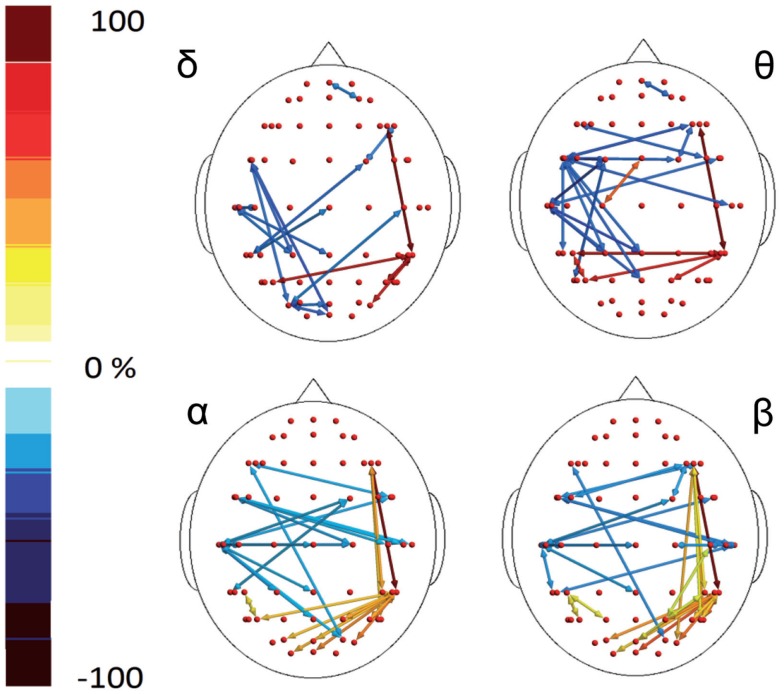
**Percent differences of SE total activity between M (migraine) vs C (controls)**. The red colors express an increase and the blue colors a reduction of SE in MIG vs CONT. For the details regarding the statistical analysis, see Tables S5 and S6 in Supplementary Material.

In the poststimulus phase, some differences were detectable between groups: while CONT showed increased synchronization between the temporal–parietal areas and the central and frontal cortical zones around the midline, migraine patients exhibited higher synchronization between the posterior parietal and the right temporal–central–frontal areas (Figures 3 and 4). For the detailed results, please refer to Tables S1–S6 in Supplementary Material.

### Analysis of Effective Connectivity: Granger Causality

The results of the GC analysis did not reveal a different behavior in the prestimulus vs poststimulus phase, while some differences emerged between the two groups in the 1 s time window following the laser stimulation.

**Figure 5:**
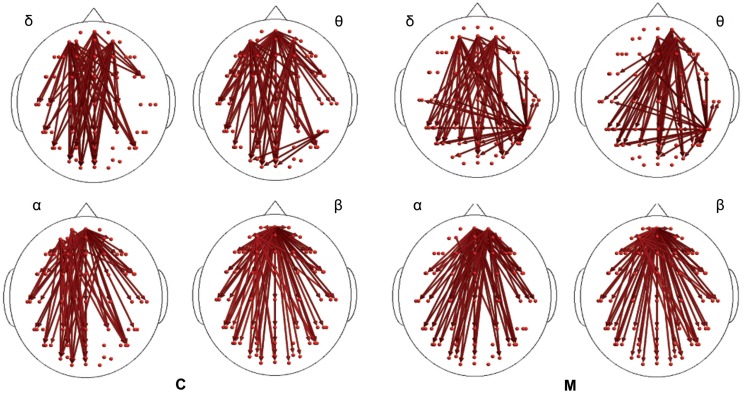
**Granger causality (GC) computed 1 s after the laser stimuli**. Statistically significant GC in main EEG maps is represented as red arrows for controls (C) and migraineurs (M) in the four bands. For the details regarding the data and statistical analysis, see Tables S7 and S8 in Supplementary Material.

**Figure 6:**
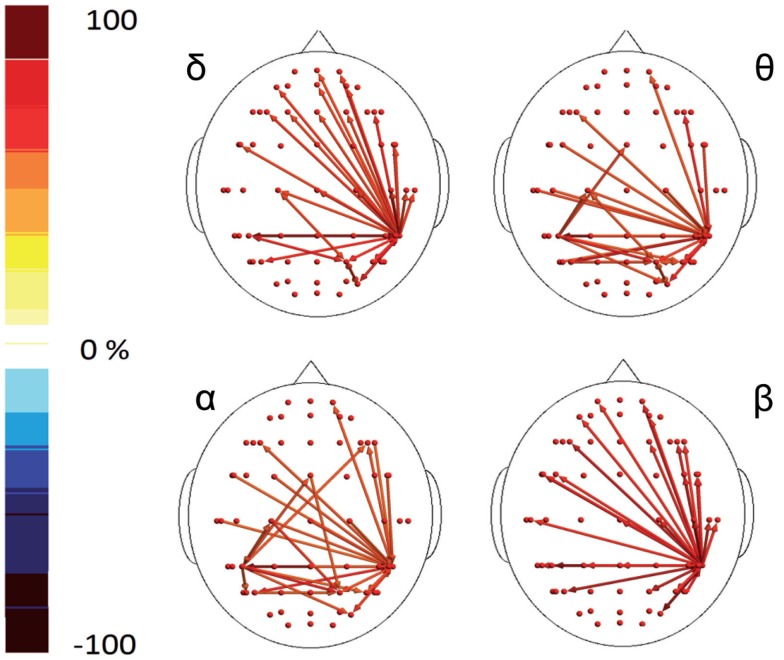
**Percent differences of GC total activity between MIG vs CONT are reported**. The red colors express an increase and the blue colors a reduction of GC in MIG vs CONT. For the details regarding the statistical analysis, see Tables S7 and S8 in Supplementary Material.

In the poststimulus phase, both groups showed a strong dynamical connection between the bilateral temporal–parietal regions and the frontal regions around the midline (Figure 5). However, in migraine patients, the areas subtended by channel CP5 and CP6 were significantly more connected with almost all the other scalp derivations, especially those in the frontal area (Figure 6). The right central–parietal zone was more connected in the “out” direction in all the considered EEG bands, while in alpha and theta bands also, the left side displayed a similar pattern (Figure 6).

For the detailed results, please refer to Tables S7 and S8 in Supplementary Material.

## Analysis of Habituation Index, Clinical Features, and GC Values’ Correlation

**Figure 7:**
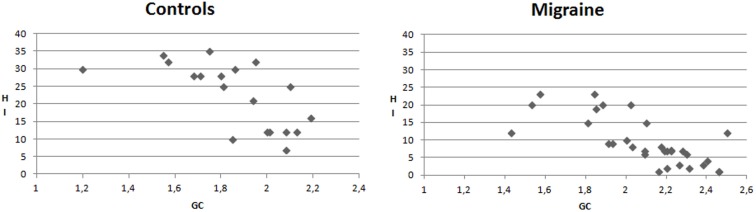
***X*–*Y* charts showing the relationship between mean values of Granger causality (GC) and Habituation index (%) of N2P2 complex in controls and migraine patients**.

In the migraine group, the mean GC values were negatively correlated with HI (Pearson correlation −0.728, *p* < 0.0001). This correlation was significant also in CONT (Pearson correlation −0.659, *p* < 0.001) (Figure 7).

The GC values were not correlated either with migraine frequency or with illness duration.

## Discussion

At the best of our knowledge, this is the first study exploring functional connectivity related to painful stimulation in migraine patients. Recent studies on resting-state functional connectivity by fMRI found that the default mode network (DMN) was different in migraine with respect to CONT, especially for the connectivity within the cortical structures promoting pain processing and modulation (Schwedt et al., 2013, 2014, 2015; Colombo et al., 2015; Liu et al., 2015; Tso et al., 2015). Most studies exploring connectivity changes preceding and following laser stimulation were performed in a small cohort of subjects implanted by subdural electrodes for treatment of epilepsy (Ohara et al., 2006; Liu et al., 2011). In those studies, attention to painful stimuli always enhanced synchrony between cortical pain-related structures. The present results suggest a different pattern of cortical connections by painful laser stimuli in migraine patients compared to CONT, using a standard recording method. Laser-evoked cortical waves are partly influenced by the degree of attention and arousal that subjects turn to pain (Lorenz and Garcia-Larrea, 2003; Iannetti et al., 2008). There are several reports suggesting that attention to pain is peculiar in migraine patients, because it is not progressively reduced during repetitive stimulation (Valeriani et al., 2003), and during contemporary cognitive tasks (de Tommaso et al., 2008). In this study, we decided to employ a standard laser-evoked potentials procedure, without specific attention manipulation, except for the warning that we routinely employ in our laboratory to reduce blinking artifacts and increase subjects’ compliance in stimuli rating. In this condition, we found that LEPs amplitude and latencies were similar between patients and CONT, though pain rating was increased in migraine. Taking into consideration that the pain threshold was similar in migraine patients and CONT, the increased pain sensitivity would be the effect of the lack of progressive cortical habituation toward painful stimulation, as frequently described in migraine patients (de Tommaso et al., 2005b). In fact in this study, we confirmed the reduction of habituation to progressive painful stimulation in migraine patients, which may be supported by a sustained attention toward stimuli during the entire stimulation task. The analysis we applied in the present study can contribute to a better understanding of pain-related cortical responses, by exploring temporal changes of EEG rhythms, which are usually hidden by the method of averaging. The wavelet analysis showed changes of EEG rhythms, especially evident in the slow bands preceding the stimulus. The same rhythms increased after the laser delivering, so the stimulus anticipation resembled the poststimulus response, according to the previous results of our group (de Tommaso et al., 2005a). In the present study, we found that the anticipatory activity appeared earlier in migraine than in CONT, confirming heightened state of readiness to pain in migraine compared to CONT (Lev et al., 2013). Enhanced anticipatory activity in the insula was also observed in other models of chronic pain as fibromyalgia or ostheoartitis, which was correlated to the severity of clinical symptoms (Brown et al., 2014). In the time preceding the laser stimulus, synchronization between the bilateral central–parietal areas and the frontal areas was enhanced in comparison with the poststimulus phase. This synchronization pattern was evident in both groups, probably expressing the effort in drawing attention after the painful stimulus warning, in accord with the previous studies describing changes in alpha rhythms synchronization before predictable painful stimuli, as a possible neural concomitant of attentive preparatory processes (Babiloni et al., 2003; Del Percio et al., 2006). Despite the fact that our analysis was applied on superficial EEG, thus not allowing for an interpretation in terms of exact source localization of cortical connections, it nonetheless suggested a synchronization between the central–parietal areas, connected to the first arrival and detection of the stimulus, and the frontal zones around the midline, which could reflect the activity of the rostral cingulate cortex, involved in arousal reaction toward pain (Peyron et al., 2000; Garcia-Larrea et al., 2003). In the studies performed with intracranial electrodes, directed attention to the painful stimulus consistently increased the degree of synchrony between primary somatosensory cortex and parasylvian regions prior to the stimulus, while the medial frontal regions were involved after the stimulus delivery (Ohara et al., 2006). Different modalities of recording as well as different ways of attention modulation may explain the different pattern of cortical synchronization preceding laser stimulus. In fact in the study by Ohara et al. (2006), patients’ attention switched from the stimulus anticipation to the stimulus counting, while in our experiment subjects were simply alerted before stimulus delivering to take attention to its features and to prevent inappropriate motor reactions. Even though manipulation of attention was different in the two studies, pain anticipation caused synchronous activation of the central–parietal areas devoted to the first steps of pain processing with the cortical frontal zones located around the midline. The central–parietal electrodes seemed the first station of functional connections, probably referring to the parietal zones of the secondary somatosensory cortex – SII – which is considered the first source of LEPs (Valeriani et al., 1996; Garcia-Larrea et al., 2003). The present analysis cannot contribute to the question regarding the origin of early LEPs, if in S1 or S2 (Valeriani et al., 2004; Valentini et al., 2012; Frot et al., 2013), though the hub of cortical connections seemed positioned in CP5 and CP6 derivations, which seem posterior in respect to S1 and more adapt to refer to the secondary somatosensory cortex. In the time following the laser stimulus delivering, we observed differences in cortical causal connections across scalp derivations between migraine patients and CONT, despite LEPs were similar for latency and amplitude. In fact, while in normal CONT the centro–parietal zones, especially of the left hemisphere, showed to be more synchronized with the omo- and contro-lateral temporal zones and the frontal zones across the midline, in migraine the same cortical derivations were more synchronized with the posterior parietal and occipital areas. Moreover, in migraine patients the centro-parietal, temporal, and frontal zones across midline, which may correspond to the main cortical sources of LEPs (e.g., somatosensory cortex, insula, and anterior cingulate), did not appear to be activated in synchrony but to be interested by a lively information transfer, specially toward the anterior midline. The biological interpretation of the application of complex algorithms to EEG rhythms derived from the scalp is approximate and somehow “philosophic.” A part from the confirmation that averaged LEPS is a simplification of complex cortical mechanisms of pain processing, which may be different in non-migraine and migraine subjects, even in the intercritical phase, we can advance some risky assumptions.

In CONT, the concurrence of increased synchronization and reduced information flow in the entire EEG spectrum, with special regard to the delta band corresponding to the averaged LEPs, may be due to the weak interactions in some neural networks devoted to pain processing, occurring during the repetitive stimulation (Pfurtscheller and Lopes da Silva, 1999). Our analysis mediated the entire EEG tracks during consecutive laser stimulation, in a time course where habituation occurred, probably inducing an “idling” rhythm involving some cortical regions in the pain network (Lei et al., 2011). In fact in healthy volunteers, synchronization occurred across the bilateral central–parietal temporal networks, with high level of GC and information transfer from the frontal regions around the midline toward the rest of the scalp derivations. Conversely, migraine patients showed increased information transfer across the bilateral – with special regard right – central–parietal temporal networks toward the bilateral frontal regions, which may be interpreted as a sign of persistent activation of these functional networks (Lei et al., 2011). The present analysis does not allow to spatially localizing the anatomic source of the cortical interconnections, though the centro-parietal networks, which seemed to be synchronized in CONT while more effectively connected in migraine patients, may refer to the first station of laser stimuli processing, as the bilateral SII and insula (Valeriani et al., 1996). The possibility that this overfunctioning of cortical connections within the pain network may correspond to LEPs dishabituation may be supposed by the correspondence between increased GC scores and reduced N2P2 habituation. However, the main source of vertex LEPs, we analyzed may not correspond to the bilateral central temporal–parietal hubs which appeared more interconnected in migraine patients and probably best represented by the early N1 component (Valeriani et al., 1996). We were not able to perform the analysis of N1 habituation, for its small amplitude and possible artifacts contamination, so we can suppose that in migraine the activation of the specific circuits devoted to laser stimulus detection may be responsible for the lack of habituation also in the following steps of pain processing, subtending the vertex LEPs. The information transfer from the frontal medial regions to the rest of scalp derivations was present in both groups, as a possible consequence of generic arousal maintenance (Legrain et al., 2011). What we presently can assume is that the LEPs amplitude variability in the course of stimulation may be explained by the dynamic changes of neuronal oscillations (Mayhew et al., 2013). Altered neural oscillations and their abnormal synchronization are crucial factors in the pathophysiology of several neuropsychiatric disorders (Fuggetta and Noh, 2013), and also migraine may be interpreted as a disorder partly caused by abnormal spontaneous thalamocortical neural oscillations, involving sensory processing and particularly pain – related neuronal networks (de Tommaso et al., 2014).

The synchronization seen in posterior parietal and occipital regions after laser stimulation in migraine may be caused by the different way of functioning of visual cortex with the possibility that idling rhythms especially in the alpha frequencies may be induced by other stimuli modalities than the visual ones (Marinazzo et al., 2008a).

The lack of significant correlation between the strength of information transfers and main migraine features, as headache frequency and age of migraine onset, may be explained by the selection of patients with episodic forms with the exclusion of chronic migraine and by the possibility that pain processing is basically different, rather than modified as a consequence of repetitive attacks. Anyway we aim to include chronic migraine for future evaluations, considering that atypical resting-state functional connectivity of affective pain-processing brain regions seemed to be correlated with disease duration (Schwedt et al., 2013), and that further synchronization and GC changes in pain networks may occur in these invalidating forms. Moreover, the abnormal dynamic connections modalities we observed in relation to decreased habituation pattern of pain-related evoked responses may be a basal feature of migraine, involving multimodal sensory processing (de Tommaso et al., 2014). This possibility may be confirmed by further studies employing multimodal stimulation tasks.

Summarizing the analysis of EEG rhythms related to painful stimulations showed differences between migraine patients and CONT during a standard stimulation procedure. Several studies confirmed a rigid pattern of laser-evoked potentials modulation in migraine patients compared to CONT, as in the condition of stimulus repetition and lack of habituation, or during distraction under cognitive tasks, with prevalent abnormalities involving the vertex complex (de Tommaso, 2008). The present brain networking model outlined an overactive nociceptive system just before stimulus delivering, with higher degree of information transfers among cortical zones involved in pain processing, which were reported to be abnormally interconnected even in the resting state (Schwedt et al., 2015). Deficient habituation may be caused by increased cortical information transfer and a different way of neuronal network oscillation, which may further suggest that a cortical dysrhythmia, probably induced by a different resonance of thalamic inputs, can contribute to differentiate normal from migraine brain.

## Author Contributions

MD substantial contributions to the conception or design of the work, acquisition, analysis, and interpretation of data for the work; drafting the work or revising it critically for important intellectual content; final approval of the version to be published; and agreement to be accountable for all aspects of the work in ensuring that questions related to the accuracy or integrity of any part of the work are appropriately investigated and resolved. GT, DM, RB, FS, and SS substantial contributions to the analysis and interpretation of data for the work; drafting the work or revising it critically for important intellectual content; final approval of the version to be published; and agreement to be accountable for all aspects of the work in ensuring that questions related to the accuracy or integrity of any part of the work are appropriately investigated and resolved. EV, KR, AM, and ML substantial contributions to the acquisition and interpretation of data for the work; drafting the work or revising it critically for important intellectual content; final approval of the version to be published; and agreement to be accountable for all aspects of the work in ensuring that questions related to the accuracy or integrity of any part of the work are appropriately investigated and resolved.

## Conflict of Interest Statement

No authors nor institution at any time received payment or services from a third party for any aspect of the submitted work. No financial relationships exist with entities that could be perceived to influence, or that give the appearance of potentially influencing, what we wrote in the submitted work. We have no patents and copyrights, whether pending, issued, licensed, and/or receiving royalties relevant to the work to declare. We have no other relationships or activities that readers could perceive to have influenced, or that give the appearance of potentially influencing, what we wrote in the submitted work.

## Footnotes

^1^http://www.antneuro.com.

^2^www.micromed-it.com.

^3^www.mathworks.com.

## References

[B1] Angelini L. de Tommaso M. Guido M. Hu K. Ivanov P. C. H. Marinazzo D. (2004). Steady-state visual evoked potentials and phase synchronization in migraine patients. Phys. Rev. Lett. 93, 038103. 10.1103/PhysRevLett.93.038103 15323876

[B2] Babiloni C. Brancucci A. Babiloni F. Capotosto P. Carducci F. Cincotti F. (2003). Anticipatory cortical responses during the expectancy of a predictable painful stimulation. A high-resolution electroencephalography study. Eur. J. Neurosci. 18, 1692–1700. 10.1046/j.1460-9568.2003.02851.x 14511347

[B3] Blinowska K. J. Kuś R. Kamiński M. (2004). Granger causality and information flow in multivariate processes. Phys. Rev. E Stat. Nonlin. Soft Matter Phys. 70, 050902. 10.1103/PhysRevE.70.050902 15600583

[B4] Brown C. A. El-Deredy W. Jones A. K. (2014). When the brain expects pain: common neural responses to pain anticipation are related to clinical pain and distress in fibromyalgia and osteoarthritis. Eur. J. Neurosci. 39, 663–672. 10.1111/ejn.12420 24219587

[B5] Colombo B. Rocca M. A. Messina R. Guerrieri S. Filippi M. (2015). Resting-state fMRI functional connectivity: a new perspective to evaluate pain modulation in migraine? Neurol. Sci. 36(Suppl. 1), 41–45. 10.1007/s10072-015-2145-x 26017510

[B6] de Tommaso M. (2008). Laser-evoked potentials in primary headaches and cranial neuralgias. Expert Rev. Neurother. 8, 1339–1345. 10.1586/14737175.8.9.1339 18759546

[B7] de Tommaso M. Ambrosini A. Brighina F. Coppola G. Perrotta A. Pierelli F. (2014). Altered processing of sensory stimuli in patients with migraine. Nat. Rev. Neurol. 10, 144–155. 10.1038/nrneurol.2014.14 24535465

[B8] de Tommaso M. Baumgartner U. Sardaro M. Difruscolo O. Serpino C. Treede R. D. (2008). Effects of distraction versus spatial discrimination on laser-evoked potentials in migraine. Headache 48, 408–416. 10.1111/j.1526-4610.2007.00857.x 18302701

[B9] de Tommaso M. Marinazzo D. Stramaglia S. (2005a). The measure of randomness by leave-one-out prediction error in the analysis of EEG after laser painful stimulation in healthy subjects and migraine patients. Clin. Neurophysiol. 116, 2775–2782. 10.1016/j.clinph.2005.08.019 16253556

[B10] de Tommaso M. Lo Sito L. Di Fruscolo O. Sardaro M. Pia Prudenzano M. Lamberti P. (2005b). Lack of habituation of nociceptive evoked responses and pain sensitivity during migraine attack. Clin. Neurophysiol. 116, 1254–1264. 10.1016/j.clinph.2005.02.018 15978487

[B11] de Tommaso M. Sciruicchio V. Ricci K. Montemurno A. Gentile F. Vecchio E. (2015). Laser-evoked potential habituation and central sensitization symptoms in childhood migraine. Cephalalgia. 10.1177/0333102415597527 26232104

[B12] de Tommaso M. Stramaglia S. Marinazzo D. Trotta G. Pellicoro M. (2013). Functional and effective connectivity in EEG alpha and beta bands during intermittent flash stimulation in migraine with and without aura. Cephalalgia 33, 938–947. 10.1177/0333102413477741 23439574

[B13] Del Percio C. Le Pera D. Arendt-Nielsen L. Babiloni C. Brancucci A. Chen A. C. (2006). Distraction affects frontal alpha rhythms related to expectancy of pain: an EEG study. Neuroimage 31, 1268–1277. 10.1016/j.neuroimage.2006.01.013 16529953

[B14] Dhamala M. Rangarajan G. Ding M. (2008). Estimating Granger causality from fourier and wavelet transforms of time series data. Phys. Rev. Lett. 100, 018701. 10.1103/PhysRevLett.100.018701 18232831

[B15] Friston K. (2011). Functional and effective connectivity: a review. Brain Connect. 1, 13–36. 10.1089/brain.2011.0008 22432952

[B16] Frot M. Magnin M. Mauguière F. Garcia-Larrea L. (2013). Cortical representation of pain in primary sensory-motor areas (S1/M1) – a study using intracortical recordings in humans. Hum. Brain Mapp. 34, 2655–2668. 10.1002/hbm.22097 22706963 PMC6869910

[B17] Fuggetta G. Noh N. A. (2013). A neurophysiological insight into the potential link between transcranial magnetic stimulation, thalamo-cortical dysrhythmia and neuropsychiatric disorders. Exp. Neurol. 245, 87–95. 10.1016/j.expneurol.2012.10.010 23063603

[B18] Garcia-Larrea L. Frot M. Valeriani M. (2003). Brain generators of laser-evoked potentials: from dipoles to functional significance. Neurophysiol. Clin. 33, 279–292. 10.1016/j.neucli.2003.10.008 14678842

[B19] Granger C. W. J. (1969). Investigating causal relations by econometric models and cross-spectral methods. Econometrica 37, 424–438. 10.2307/1912791

[B20] Hashmi J. A. Kong J. Spaeth R. Khan S. Kaptchuk T. J. Gollub R. L. (2014). Functional network architecture predicts psychologically mediated analgesia related to treatment in chronic knee pain patients. J. Neurosci. 34, 3924–3936. 10.1523/JNEUROSCI.3155-13.2014 24623770 PMC3951694

[B21] Headache Classification Committee. (2004). The international classification of headache disorders II. Cephalalgia 24, 9–160. 10.1111/j.1468-2982.2004.00653 14979299

[B22] Iannetti G. D. Hughes N. P. Lee M. C. Mouraux A. (2008). Determinants of laser-evoked EEG responses: pain perception or stimulus saliency? J. Neurophysiol. 100, 815–828. 10.1152/jn.00097.2008 18525021 PMC2525705

[B23] Legrain V. Iannetti G. D. Plaghki L. Mouraux A. (2011). The pain matrix reloaded: a salience detection system for the body. Prog. Neurobiol. 93, 111–124. 10.1016/j.pneurobio.2010.10.005 21040755

[B24] Lei X. Ostwald D. Hu J. Qiu C. Porcaro C. Bagshaw A. P. (2011). Multimodal functional network connectivity: an EEG-fMRI fusion in network space. PLoS ONE 6:e24642. 10.1371/journal.pone.0024642 21961040 PMC3178514

[B25] Lev R. Granovsky Y. Yarnitsky D. (2013). Enhanced pain expectation in migraine: EEG-based evidence for impaired prefrontal function. Headache 53, 1054–1070. 10.1111/j.1526-4610.2012.02297.x 23216259

[B26] Liu C. C. Crone N. E. Franaszczuk P. J. Cheng D. T. Schretlen D. S. Lenz F. A. (2011). Fear conditioning is associated with dynamic directed functional interactions between and within the human amygdala, hippocampus, and frontal lobe. Neuroscience 189, 359–369. 10.1016/j.neuroscience.2011.05.067 21664438 PMC3150454

[B27] Liu J. Zhao L. Lei F. Zhang Y. Yuan K. Gong Q. (2015). Disrupted resting-state functional connectivity and its changing trend in migraine suffers. Hum. Brain Mapp. 36, 1892–1907. 10.1002/hbm.22744 25640857 PMC6869678

[B28] Lorenz J. Garcia-Larrea L. (2003). Contribution of attentional and cognitive factors to laser evoked brain potentials. Neurophysiol. Clin. 33, 293–301. 10.1016/j.neucli.2003.10.004 14678843

[B29] Louter M. A. Bosker J. E. Van Oosterhout W. P. Van Zwet E. W. Zitman F. G. Ferrari M. D. (2013). Cutaneous allodynia as a predictor of migraine chronification. Brain 136(Pt 11), 3489–3496. 10.1093/brain/awt251 24080152

[B30] Marinazzo D. Pellicoro M. Stramaglia S. (2008a). Kernel Granger causality and the analysis of dynamical networks. Phys. Rev. E Stat. Nonlin. Soft Matter Phys. 77, 056215 10.1103/PhysRevE.77.056215 18643150

[B31] Marinazzo D. Pellicoro M. Stramaglia S. (2008b). Kernel method for nonlinear Granger causality. Phys. Rev. Lett. 100, 144103 10.1103/PhysRevLett.100.144103 18518037

[B32] Mayhew S. D. Hylands-White N. Porcaro C. Derbyshire S. W. Bagshaw A. P. (2013). Intrinsic variability in the human response to pain is assembled from multiple, dynamic brain processes. Neuroimage 15, 68–78. 10.1016/j.neuroimage.2013.02.028 23485593

[B33] Ohara S. Crone N. E. Weiss N. Lenz F. A. (2006). Analysis of synchrony demonstrates ‘pain networks’ defined by rapidly switching, task-specific, functional connectivity between pain-related cortical structures. Pain 123, 244–253. 10.1016/j.pain.2006.02.012 16563627

[B34] Ohara S. Croneb N. E. Weissa N. Lenz F. (2004). Attention to a painful cutaneous laser stimulus modulates electrocorticographic event-related desynchronization in humans. Neurophysiol. Clin. 115, 1641–1652. 10.1016/j.clinph.2004.02.023 15203065

[B35] Peyron R. Laurent B. García-Larrea L. (2000). Functional imaging of brain responses to pain. A review and meta-analysis. Neurophysiol. Clin. 30, 263–288. 10.1016/S0987-7053(00)00227-6 11126640

[B36] Pfurtscheller G. Lopes da Silva F. H. (1999). Event-related EEG/MEG synchronization and desynchronization: basic principles. Clin. Neurophysiol. 110, 1842–1857. 10.1016/S1388-2457(99)00141-8 10576479

[B37] Schwedt T. J. Chiang C. C. Chong C. D. Dodick D. W. (2015). Functional MRI of migraine. Lancet Neurol. 14, 81–91. 10.1016/S1474-4422(14)70193-0 25496899 PMC11318354

[B38] Schwedt T. J. Larson-Prior L. Coalson R. S. Nolan T. Mar S. Ances B. M. (2014). Allodynia and descending pain modulation in migraine: a resting state functional connectivity analysis. Pain Med. 15, 154–165. 10.1111/pme.12267 24165094 PMC4188437

[B39] Schwedt T. J. Schlaggar B. L. Mar S. Nolan T. Coalson R. S. Nardos B. (2013). Atypical resting-state functional connectivity of affective pain regions in chronic migraine. Headache 53, 737–751. 10.1111/head.12081 23551164 PMC3637407

[B40] Tass P. Rosenblum M. G. Weule J. Kurths J. Pikovsky A. Volkmann J. (1998). Detection of n:m phase locking from noisy data: application to magnetoencephalography. Phys. Rev. Lett. 81, 3291–3294. 10.1103/PhysRevLett.81.3291

[B41] Treede R. D. Lorenz J. Baumgärtner U. (2003). Clinical usefulness of laser-evoked potentials. Neurophysiol. Clin. 33, 303–314. 10.1016/j.neucli.2003.10.009 14678844

[B42] Tso A. R. Trujillo A. Guo C. C. Goadsby P. J. Seeley W. W. (2015). The anterior insula shows heightened interictal intrinsic connectivity in migraine without aura. Neurology 84, 1043–1050. 10.1212/WNL.0000000000001330 25663219 PMC4352101

[B43] Valentini E. Hu L. Chakrabarti B. Hu Y. Aglioti S. M. Iannetti G. D. (2012). The primary somatosensory cortex largely contributes to the early part of the cortical response elicited by nociceptive stimuli. Neuroimage 59, 1571–1581. 10.1016/j.neuroimage.2011.08.069 21906686

[B44] Valeriani M. Barba C. Le Pera D. Restuccia D. Colicchio G. Tonali P. (2004). Different neuronal contribution to N20 somatosensory evoked potential and to CO2 laser evoked potentials: an intracerebral recording study. Clin. Neurophysiol. 115, 211–216. 10.1016/S1388-2457(03)00287-6 14706490

[B45] Valeriani M. de Tommaso M. Restuccia D. Le Pera D. Guido M. Iannetti G. D. (2003). Reduced habituation to experimental pain in migraine patients: a CO(2) laser evoked potential study. Pain 105, 57–64. 10.1016/S0304-3959(03)00137-4 14499420

[B46] Valeriani M. Rambaud L. Mauguière F. (1996). Scalp topography and dipolar source modelling of potentials evoked by CO2 laser stimulation of the hand. Electroencephalogr. Clin. Neurophysiol. 100, 343–353. 10.1016/0168-5597(96)95625-7 17441304

