# Supplementary material for: Functional Connectivity of EEG Signals Under Laser Stimulation in Migraine
Source: Front Hum Neurosci. 2015 Nov 24;9:640. doi: 10.3389/fnhum.2015.00640 (PMC4656845; doi:10.3389/fnhum.2015.00640)
Supplement: Supplementary file 1 [file Table_1.DOCX]

| delta |  |  |  |  | theta |  |  |  |  |
| --- | --- | --- | --- | --- | --- | --- | --- | --- | --- |
| Couples | PRE (bits) | POST (bits) | Percentual  difference | Corrected  ANOVA  p-value | Couples | PRE (bits) | POST (bits) | Percentual  difference | Corrected ANOVA  p-value |
| FP1-PO7 | 0,204 | 0,180 | 13 | 0,012 | FPZ-T6 | 0,198 | 0,174 | 13 | 0,002 |
| FPZ-T5 | 0,223 | 0,199 | 12 | 0,014 | FZ-O1 | 0,193 | 0,172 | 12 | 0,010 |
| FPZ-OZ | 0,216 | 0,193 | 12 | 0,016 | FZ-P6 | 0,203 | 0,178 | 13 | 0,001 |
| FPZ-TP7 | 0,223 | 0,198 | 12 | 0,009 | FZ-PO8 | 0,188 | 0,167 | 12 | 0,015 |
| FP2-FZ | 0,213 | 0,187 | 13 | 0,013 | F4-T6 | 0,199 | 0,176 | 13 | 0,006 |
| FP2-F4 | 0,215 | 0,187 | 14 | 0,011 | PZ-AF4 | 0,196 | 0,175 | 12 | 0,008 |
| FP2-PZ | 0,201 | 0,175 | 14 | 0,014 | T6-FPZ | 0,198 | 0,174 | 13 | 0,002 |
| FP2-OZ | 0,206 | 0,174 | 17 | 0,002 | T6-F4 | 0,199 | 0,176 | 13 | 0,006 |
| FP2-O2 | 0,199 | 0,171 | 15 | 0,012 | T6-FC2 | 0,199 | 0,175 | 13 | 0,003 |
| FP2-F5 | 0,209 | 0,178 | 16 | 0,003 | T6-FT7 | 0,197 | 0,172 | 13 | 0,009 |
| FP2-PO7 | 0,201 | 0,167 | 18 | 0,000 | O1-FZ | 0,193 | 0,172 | 12 | 0,010 |
| FP2-POZ | 0,201 | 0,173 | 15 | 0,005 | O1-O2 | 0,181 | 0,158 | 14 | 0,010 |
| F3-O1 | 0,207 | 0,179 | 14 | 0,008 | O1-FC5 | 0,200 | 0,178 | 12 | 0,010 |
| F3-OZ | 0,209 | 0,180 | 15 | 0,005 | O1-F1 | 0,191 | 0,170 | 12 | 0,008 |
| F3-CP2 | 0,217 | 0,182 | 17 | 0,001 | O1-FC4 | 0,193 | 0,172 | 12 | 0,006 |
| F3-PO3 | 0,202 | 0,172 | 16 | 0,005 | F6-AF4 | 0,185 | 0,163 | 13 | 0,008 |
| F3-PO7 | 0,211 | 0,183 | 14 | 0,010 | O2-O1 | 0,181 | 0,158 | 14 | 0,010 |
| FZ-FP2 | 0,213 | 0,187 | 13 | 0,013 | FC2-T6 | 0,199 | 0,175 | 13 | 0,003 |
| F4-FP2 | 0,215 | 0,187 | 14 | 0,011 | FC2-CP5 | 0,206 | 0,183 | 12 | 0,007 |
| CZ-C2 | 0,258 | 0,282 | 10 | 0,014 | FC2-POZ | 0,204 | 0,182 | 12 | 0,010 |
| C4-CP1 | 0,234 | 0,262 | 13 | 0,007 | FC5-O1 | 0,200 | 0,178 | 12 | 0,010 |
| T5-FPZ | 0,223 | 0,199 | 12 | 0,014 | FC5-FT8 | 0,199 | 0,174 | 14 | 0,003 |
| PZ-FP2 | 0,201 | 0,175 | 14 | 0,014 | CP5-FC2 | 0,206 | 0,183 | 12 | 0,007 |
| O1-F3 | 0,207 | 0,179 | 14 | 0,008 | CP5-CP6 | 0,195 | 0,172 | 13 | 0,002 |
| OZ-FPZ | 0,216 | 0,193 | 12 | 0,016 | CP6-CP5 | 0,195 | 0,172 | 13 | 0,002 |
| OZ-FP2 | 0,206 | 0,174 | 17 | 0,002 | AF4-PZ | 0,196 | 0,175 | 12 | 0,008 |
| OZ-F3 | 0,209 | 0,180 | 15 | 0,005 | AF4-F6 | 0,185 | 0,163 | 13 | 0,008 |
| OZ-F1 | 0,220 | 0,196 | 12 | 0,013 | F1-O1 | 0,191 | 0,170 | 12 | 0,008 |
| OZ-C5 | 0,230 | 0,206 | 11 | 0,012 | FT7-T6 | 0,197 | 0,172 | 13 | 0,009 |
| O2-FP2 | 0,199 | 0,171 | 15 | 0,012 | FT7-FT8 | 0,185 | 0,162 | 14 | 0,006 |
| CP1-C4 | 0,234 | 0,262 | 13 | 0,007 | FC4-O1 | 0,193 | 0,172 | 12 | 0,006 |
| CP1-FC4 | 0,216 | 0,240 | 12 | 0,012 | FT8-FC5 | 0,199 | 0,174 | 14 | 0,003 |
| CP2-F3 | 0,217 | 0,182 | 17 | 0,001 | FT8-FT7 | 0,185 | 0,162 | 14 | 0,006 |
| CP2-AFZ | 0,223 | 0,199 | 12 | 0,012 | P6-FZ | 0,203 | 0,178 | 13 | 0,001 |
| PO3-F3 | 0,202 | 0,172 | 16 | 0,005 | POZ-FC2 | 0,204 | 0,182 | 12 | 0,010 |
| AFZ-CP2 | 0,223 | 0,199 | 12 | 0,012 | PO8-FZ | 0,188 | 0,167 | 12 | 0,015 |
| AFZ-PO7 | 0,221 | 0,197 | 12 | 0,014 | AFZ-PO7 | 0,221 | 0,197 | 12 | 0,014 |
| F5-FP2 | 0,209 | 0,178 | 16 | 0,003 | F5-FP2 | 0,209 | 0,178 | 16 | 0,003 |
| F1-OZ | 0,220 | 0,196 | 12 | 0,013 | F1-OZ | 0,220 | 0,196 | 12 | 0,013 |
| FC3-P5 | 0,221 | 0,191 | 15 | 0,003 | FC3-P5 | 0,221 | 0,191 | 15 | 0,003 |
| FC3-PO7 | 0,222 | 0,197 | 12 | 0,008 | FC3-PO7 | 0,222 | 0,197 | 12 | 0,008 |
| FC4-CP1 | 0,216 | 0,240 | 12 | 0,012 | FC4-CP1 | 0,216 | 0,240 | 12 | 0,012 |
| C5-OZ | 0,230 | 0,206 | 11 | 0,012 | C5-OZ | 0,230 | 0,206 | 11 | 0,012 |
| C2-CZ | 0,258 | 0,282 | 10 | 0,014 | C2-CZ | 0,258 | 0,282 | 10 | 0,014 |
| TP7-FPZ | 0,223 | 0,198 | 12 | 0,009 | TP7-FPZ | 0,223 | 0,198 | 12 | 0,009 |
| P5-FC3 | 0,221 | 0,191 | 15 | 0,003 | P5-FC3 | 0,221 | 0,191 | 15 | 0,003 |
| PO7-FP1 | 0,204 | 0,180 | 13 | 0,012 | PO7-FP1 | 0,204 | 0,180 | 13 | 0,012 |
| PO7-FP2 | 0,201 | 0,167 | 18 | 0,000 | PO7-FP2 | 0,201 | 0,167 | 18 | 0,000 |
| PO7-F3 | 0,211 | 0,183 | 14 | 0,010 | PO7-F3 | 0,211 | 0,183 | 14 | 0,010 |
| PO7-AFZ | 0,221 | 0,197 | 12 | 0,014 | PO7-AFZ | 0,221 | 0,197 | 12 | 0,014 |
| PO7-FC3 | 0,222 | 0,197 | 12 | 0,008 | PO7-FC3 | 0,222 | 0,197 | 12 | 0,008 |
| POZ-FP2 | 0,201 | 0,173 | 15 | 0,005 | POZ-FP2 | 0,201 | 0,173 | 15 | 0,005 |

Table 1-S – Synchronization Entropy: the most significant differences between Pre and Post conditions in CONT; results by ANOVA test with the Bonferroni-Holmes correction for delta band and theta bands are shown.
