# Supplementary material for: Functional Connectivity of EEG Signals Under Laser Stimulation in Migraine
Source: Front Hum Neurosci. 2015 Nov 24;9:640. doi: 10.3389/fnhum.2015.00640 (PMC4656845; doi:10.3389/fnhum.2015.00640)
Supplement: Supplementary file 2 [file Table_2.DOCX]

| alpha |  |  |  |  | beta |  |  |  |  |
| --- | --- | --- | --- | --- | --- | --- | --- | --- | --- |
| Couples | PRE (bits) | POST (bits) | Percentual difference | Corrected  ANOVA  p-value | Couples | PRE (bits) | POST (bits) | Percentual difference | Corrected  ANOVA  p-value |
| FP1-C4 | 0,149 | 0,129 | 14 | 0,006 | FZ-CPZ | 0,139 | 0,153 | 11 | 0,014 |
| FP1-P6 | 0,143 | 0,126 | 13 | 0,011 | C3-C2 | 0,177 | 0,190 | 9 | 0,014 |
| F7-F8 | 0,154 | 0,139 | 11 | 0,014 | C2-C3 | 0,177 | 0,190 | 9 | 0,014 |
| F7-P6 | 0,159 | 0,142 | 11 | 0,009 | CPZ-FZ | 0,139 | 0,153 | 11 | 0,014 |
| F8-F7 | 0,154 | 0,139 | 11 | 0,014 | F8-F7 | 0,154 | 0,139 | 11 | 0,014 |
| C3-C5 | 0,217 | 0,241 | 12 | 0,003 | C3-C5 | 0,217 | 0,241 | 12 | 0,003 |
| CZ-O1 | 0,171 | 0,154 | 11 | 0,008 | CZ-O1 | 0,171 | 0,154 | 11 | 0,008 |
| C4-FP1 | 0,149 | 0,129 | 14 | 0,006 | C4-FP1 | 0,149 | 0,129 | 14 | 0,006 |
| C4-T6 | 0,185 | 0,168 | 10 | 0,015 | C4-T6 | 0,185 | 0,168 | 10 | 0,015 |
| T6-C4 | 0,185 | 0,168 | 10 | 0,015 | T6-C4 | 0,185 | 0,168 | 10 | 0,015 |
| T6-C6 | 0,201 | 0,183 | 10 | 0,016 | T6-C6 | 0,201 | 0,183 | 10 | 0,016 |
| O1-CZ | 0,171 | 0,154 | 11 | 0,008 | O1-CZ | 0,171 | 0,154 | 11 | 0,008 |
| O1-FC1 | 0,201 | 0,172 | 16 | 0,000 | O1-FC1 | 0,201 | 0,172 | 16 | 0,000 |
| O1-CP1 | 0,188 | 0,169 | 11 | 0,006 | O1-CP1 | 0,188 | 0,169 | 11 | 0,006 |
| O1-C1 | 0,168 | 0,148 | 13 | 0,009 | O1-C1 | 0,168 | 0,148 | 13 | 0,009 |
| O1-P1 | 0,215 | 0,195 | 10 | 0,009 | O1-P1 | 0,215 | 0,195 | 10 | 0,009 |
| OZ-FC1 | 0,194 | 0,176 | 10 | 0,013 | OZ-FC1 | 0,194 | 0,176 | 10 | 0,013 |
| OZ-P1 | 0,216 | 0,197 | 10 | 0,016 | OZ-P1 | 0,216 | 0,197 | 10 | 0,016 |
| F6-FC2 | 0,152 | 0,135 | 12 | 0,015 | F6-FC2 | 0,152 | 0,135 | 12 | 0,015 |
| FC2-F6 | 0,152 | 0,135 | 12 | 0,015 | FC2-F6 | 0,152 | 0,135 | 12 | 0,015 |
| FC1-O1 | 0,201 | 0,172 | 16 | 0,000 | FC1-O1 | 0,201 | 0,172 | 16 | 0,000 |
| FC1-OZ | 0,194 | 0,176 | 10 | 0,013 | FC1-OZ | 0,194 | 0,176 | 10 | 0,013 |
| FC1-CP2 | 0,213 | 0,189 | 12 | 0,002 | FC1-CP2 | 0,213 | 0,189 | 12 | 0,002 |
| FC1-C5 | 0,194 | 0,213 | 11 | 0,010 | FC1-C5 | 0,194 | 0,213 | 11 | 0,010 |
| FC1-PO7 | 0,204 | 0,186 | 10 | 0,013 | FC1-PO7 | 0,204 | 0,186 | 10 | 0,013 |
| CP1-O1 | 0,188 | 0,169 | 11 | 0,006 | CP1-O1 | 0,188 | 0,169 | 11 | 0,006 |
| CP2-FC1 | 0,213 | 0,189 | 12 | 0,002 | CP2-FC1 | 0,213 | 0,189 | 12 | 0,002 |
| CP2-CPZ | 0,215 | 0,196 | 10 | 0,013 | CP2-CPZ | 0,215 | 0,196 | 10 | 0,013 |
| FC5-CP6 | 0,187 | 0,167 | 12 | 0,006 | FC5-CP6 | 0,187 | 0,167 | 12 | 0,006 |
| CP6-FC5 | 0,187 | 0,167 | 12 | 0,006 | CP6-FC5 | 0,187 | 0,167 | 12 | 0,006 |
| CP6-AF7 | 0,165 | 0,147 | 12 | 0,006 | CP6-AF7 | 0,165 | 0,147 | 12 | 0,006 |
| CP6-F2 | 0,158 | 0,142 | 11 | 0,007 | CP6-F2 | 0,158 | 0,142 | 11 | 0,007 |
| CP6-FC4 | 0,170 | 0,153 | 11 | 0,015 | CP6-FC4 | 0,170 | 0,153 | 11 | 0,015 |
| AF7-CP6 | 0,165 | 0,147 | 12 | 0,006 | AF7-CP6 | 0,165 | 0,147 | 12 | 0,006 |
| AF4-AF8 | 0,154 | 0,138 | 12 | 0,008 | AF4-AF8 | 0,154 | 0,138 | 12 | 0,008 |
| AF8-AF4 | 0,154 | 0,138 | 12 | 0,008 | AF8-AF4 | 0,154 | 0,138 | 12 | 0,008 |
| F2-CP6 | 0,158 | 0,142 | 11 | 0,007 | F2-CP6 | 0,158 | 0,142 | 11 | 0,007 |
| FC4-CP6 | 0,170 | 0,153 | 11 | 0,015 | FC4-CP6 | 0,170 | 0,153 | 11 | 0,015 |
| C5-C3 | 0,217 | 0,241 | 12 | 0,003 | C5-C3 | 0,217 | 0,241 | 12 | 0,003 |
| C5-FC1 | 0,194 | 0,213 | 11 | 0,010 | C5-FC1 | 0,194 | 0,213 | 11 | 0,010 |
| C1-O1 | 0,168 | 0,148 | 13 | 0,009 | C1-O1 | 0,168 | 0,148 | 13 | 0,009 |
| C6-T6 | 0,201 | 0,183 | 10 | 0,016 | C6-T6 | 0,201 | 0,183 | 10 | 0,016 |
| CPZ-CP2 | 0,215 | 0,196 | 10 | 0,013 | CPZ-CP2 | 0,215 | 0,196 | 10 | 0,013 |
| P1-O1 | 0,215 | 0,195 | 10 | 0,009 | P1-O1 | 0,215 | 0,195 | 10 | 0,009 |
| P1-OZ | 0,216 | 0,197 | 10 | 0,016 | P1-OZ | 0,216 | 0,197 | 10 | 0,016 |
| P6-FP1 | 0,143 | 0,126 | 13 | 0,011 | P6-FP1 | 0,143 | 0,126 | 13 | 0,011 |
| P6-F7 | 0,159 | 0,142 | 11 | 0,009 | P6-F7 | 0,159 | 0,142 | 11 | 0,009 |
| PO7-FC1 | 0,204 | 0,186 | 10 | 0,013 | PO7-FC1 | 0,204 | 0,186 | 10 | 0,013 |
| PO7-F3 | 0,211 | 0,183 | 14 | 0,010 | PO7-F3 | 0,211 | 0,183 | 14 | 0,010 |
| PO7-AFZ | 0,221 | 0,197 | 12 | 0,014 | PO7-AFZ | 0,221 | 0,197 | 12 | 0,014 |
| PO7-FC3 | 0,222 | 0,197 | 12 | 0,008 | PO7-FC3 | 0,222 | 0,197 | 12 | 0,008 |
| POZ-FP2 | 0,201 | 0,173 | 15 | 0,005 | POZ-FP2 | 0,201 | 0,173 | 15 | 0,005 |

Table 2-S – Synchronization Entropy: the most significant differences between Pre and Post conditions in controls (CONT); results by ANOVA test with the Bonferroni-Holmes correction for alpha band and beta bands are shown.
