# Supplementary material for: Functional Connectivity of EEG Signals Under Laser Stimulation in Migraine
Source: Front Hum Neurosci. 2015 Nov 24;9:640. doi: 10.3389/fnhum.2015.00640 (PMC4656845; doi:10.3389/fnhum.2015.00640)
Supplement: Supplementary file 3 [file Table_3.DOCX]

| delta |  |  |  |  | theta |  |  |  |  |
| --- | --- | --- | --- | --- | --- | --- | --- | --- | --- |
| Couples | PRE (bits) | POST (bits) | Percentual difference | Corrected  ANOVA  p-value | Couples | PRE (bits) | POST (bits) | Percentual difference | Corrected  ANOVA  p-value |
| FP2-FT7 | 0,19000 | 0,169 | 12 | 0,00754 | FPZ-O2 | 0,177 | 0,195 | 11 | 0,00695 |
| FZ-CP2 | 0,18600 | 0,204 | 11 | 0,00837 | FPZ-FC2 | 0,164 | 0,183 | 12 | 0,00591 |
| FZ-AF3 | 0,21700 | 0,24 | 12 | 0,00102 | F7-TP7 | 0,175 | 0,194 | 12 | 0,00306 |
| F4-F5 | 0,18400 | 0,204 | 12 | 0,00484 | F3-CPZ | 0,176 | 0,193 | 11 | 0,00587 |
| C3-FC6 | 0,20900 | 0,23 | 11 | 0,00390 | F3-P1 | 0,175 | 0,193 | 11 | 0,00532 |
| C3-AFZ | 0,20400 | 0,226 | 12 | 0,00193 | F4-AFZ | 0,171 | 0,191 | 12 | 0,00096 |
| C3-FC3 | 0,22900 | 0,252 | 11 | 0,00159 | T3-TP7 | 0,204 | 0,224 | 11 | 0,00677 |
| C3-C2 | 0,21500 | 0,237 | 11 | 0,00205 | C3-CPZ | 0,212 | 0,236 | 12 | 0,00037 |
| C4-C5 | 0,19600 | 0,223 | 14 | 0,00024 | C4-PZ | 0,203 | 0,224 | 11 | 0,00149 |
| C4-PO7 | 0,18400 | 0,207 | 14 | 0,00139 | PZ-C4 | 0,203 | 0,224 | 11 | 0,00149 |
| P3-PZ | 0,22000 | 0,242 | 11 | 0,00337 | PZ-CP1 | 0,225 | 0,249 | 12 | 0,00081 |
| P3-C5 | 0,20800 | 0,233 | 13 | 0,00071 | PZ-CP4 | 0,201 | 0,224 | 12 | 0,00147 |
| PZ-P3 | 0,22000 | 0,242 | 11 | 0,00337 | O2-FPZ | 0,177 | 0,195 | 11 | 0,00695 |
| PZ-FCZ | 0,20600 | 0,228 | 12 | 0,00251 | FC2-FPZ | 0,164 | 0,183 | 12 | 0,00591 |
| FC2-CP3 | 0,19400 | 0,214 | 11 | 0,01575 | FC1-CPZ | 0,223 | 0,246 | 11 | 0,00201 |
| CP2-FZ | 0,18600 | 0,204 | 11 | 0,00837 | FC1-CP4 | 0,185 | 0,205 | 12 | 0,00498 |
| CP2-C1 | 0,20400 | 0,224 | 11 | 0,00246 | CP1-PZ | 0,225 | 0,249 | 12 | 0,00081 |
| FC6-C3 | 0,20900 | 0,23 | 11 | 0,00390 | AFZ-F4 | 0,171 | 0,191 | 12 | 0,00096 |
| AFZ-C3 | 0,20400 | 0,226 | 12 | 0,00193 | C5-CP4 | 0,170 | 0,189 | 12 | 0,00344 |
| AF3-FZ | 0,21700 | 0,24 | 12 | 0,00102 | C1-CPZ | 0,217 | 0,238 | 11 | 0,00157 |
| F5-F4 | 0,18400 | 0,204 | 12 | 0,00484 | TP7-F7 | 0,175 | 0,194 | 12 | 0,00306 |
| F5-FC4 | 0,19200 | 0,214 | 12 | 0,00313 | TP7-T3 | 0,204 | 0,224 | 11 | 0,00677 |
| FT7-FP2 | 0,19000 | 0,169 | 12 | 0,00754 | CP3-CPZ | 0,201 | 0,224 | 12 | 0,00047 |
| FC3-C3 | 0,22900 | 0,252 | 11 | 0,00159 | CPZ-F3 | 0,176 | 0,193 | 11 | 0,00587 |
| FCZ-PZ | 0,20600 | 0,228 | 12 | 0,00251 | CPZ-C3 | 0,212 | 0,236 | 12 | 0,00037 |
| FC4-F5 | 0,19200 | 0,214 | 12 | 0,00313 | CPZ-FC1 | 0,223 | 0,246 | 11 | 0,00201 |
| C5-C4 | 0,19600 | 0,223 | 14 | 0,00024 | CPZ-C1 | 0,217 | 0,238 | 11 | 0,00157 |
| C5-P3 | 0,20800 | 0,233 | 13 | 0,00071 | CPZ-CP3 | 0,201 | 0,224 | 12 | 0,00047 |
| C5-CP4 | 0,18800 | 0,207 | 11 | 0,00872 | CPZ-CP4 | 0,200 | 0,224 | 13 | 0,00053 |
| C5-PO8 | 0,18800 | 0,208 | 12 | 0,00304 | CP4-PZ | 0,201 | 0,224 | 12 | 0,00147 |
| C1-CP2 | 0,20400 | 0,224 | 11 | 0,00246 | CP4-FC1 | 0,185 | 0,205 | 12 | 0,00498 |
| C2-C3 | 0,21500 | 0,237 | 11 | 0,00205 | CP4-C5 | 0,170 | 0,189 | 12 | 0,00344 |
| CP3-FC2 | 0,19400 | 0,214 | 11 | 0,01575 | CP4-CPZ | 0,200 | 0,224 | 13 | 0,00053 |
| CP4-C5 | 0,18800 | 0,207 | 11 | 0,00872 | CP4-P2 | 0,199 | 0,221 | 12 | 0,00744 |
| PO7-C4 | 0,18400 | 0,207 | 14 | 0,00139 | P1-F3 | 0,175 | 0,193 | 11 | 0,00532 |
| PO8-C5 | 0,18800 | 0,208 | 12 | 0,00304 | P2-CP4' | 0,199 | 0,221 | 12 | 0,00744 |

Table 3-S – Synchronization Entropy: the most significant differences between Pre and Post conditions in Migraine patients; results by ANOVA test with the Bonferroni-Holmes correction for delta band and theta bands are shown.
