# Supplementary material for: Functional Connectivity of EEG Signals Under Laser Stimulation in Migraine
Source: Front Hum Neurosci. 2015 Nov 24;9:640. doi: 10.3389/fnhum.2015.00640 (PMC4656845; doi:10.3389/fnhum.2015.00640)
Supplement: Supplementary file 4 [file Table_4.DOCX]

| alpha |  |  |  |  | beta |  |  |  |  |
| --- | --- | --- | --- | --- | --- | --- | --- | --- | --- |
| Couples | PRE (bits) | POST (bits) | Percentual difference | Corrected  ANOVA  p-value | Couples | PRE (bits) | POST (bits) | Percentual difference | Corrected  ANOVA  p-value |
| C4-FC1 | 0,172 | 0,192 | 12 | 0,00092 | C3-CZ | 0,182 | 0,193 | 7 | 0,01590 |
| C4-C1 | 0,178 | 0,199 | 13 | 0,00022 | C3-C4 | 0,113 | 0,122 | 9 | 0,01192 |
| C4-CPZ | 0,196 | 0,219 | 12 | 0,00023 | C3-CP1 | 0,134 | 0,147 | 10 | 0,00114 |
| P3-PO7 | 0,187 | 0,214 | 16 | 4,083E-05 | C3-C2 | 0,139 | 0,151 | 9 | 0,00569 |
| PZ-P1 | 0,227 | 0,252 | 12 | 0,00090 | C3-CPZ | 0,176 | 0,187 | 7 | 0,00742 |
| O1-F6 | 0,192 | 0,214 | 12 | 0,00068 | CZ-C3 | 0,182 | 0,193 | 7 | 0,01590 |
| O1-PO8 | 0,172 | 0,197 | 16 | 0,00002 | CZ-CP1 | 0,203 | 0,216 | 7 | 0,00489 |
| OZ-PO3 | 0,201 | 0,224 | 13 | 0,00233 | CZ-CPZ | 0,241 | 0,257 | 8 | 0,00117 |
| F6-O1 | 0,192 | 0,214 | 12 | 0,00068 | C4-C3 | 0,113 | 0,122 | 9 | 0,01192 |
| F6-PO7 | 0,165 | 0,186 | 14 | 0,00025 | PZ-FC2 | 0,117 | 0,128 | 10 | 0,01300 |
| FC1-C4 | 0,172 | 0,192 | 12 | 0,00092 | PZ-FC3 | 0,116 | 0,125 | 9 | 0,01333 |
| FC1-P2 | 0,182 | 0,205 | 14 | 0,00078 | FC2-PZ | 0,117 | 0,128 | 10 | 0,01300 |
| CP1-CPZ | 0,225 | 0,249 | 12 | 0,00039 | FC2-CP1 | 0,117 | 0,129 | 11 | 0,00332 |
| CP2-PO8 | 0,172 | 0,191 | 12 | 0,00023 | CP1-C3 | 0,134 | 0,147 | 10 | 0,00114 |
| PO3-OZ | 0,201 | 0,224 | 13 | 0,00233 | CP1-CZ | 0,203 | 0,216 | 7 | 0,00489 |
| F1-CP3 | 0,143 | 0,158 | 12 | 0,00057 | CP1-FC2 | 0,117 | 0,129 | 11 | 0,00332 |
| C5-TP8 | 0,142 | 0,157 | 12 | 0,00253 | CP1-FC3 | 0,108 | 0,117 | 9 | 0,01175 |
| C5-P2 | 0,154 | 0,17 | 12 | 0,00177 | F1-POZ | 0,082 | 0,0888 | 10 | 0,01542 |
| C5-PO8 | 0,147 | 0,163 | 12 | 0,00041 | FC3-PZ | 0,116 | 0,125 | 9 | 0,01333 |
| C1-C4 | 0,178 | 0,199 | 13 | 0,00022 | FC3-CP1 | 0,108 | 0,117 | 9 | 0,01175 |
| C6-CP3 | 0,151 | 0,167 | 12 | 0,00084 | C2-C3 | 0,139 | 0,151 | 9 | 0,00569 |
| C6-CPZ | 0,166 | 0,185 | 13 | 0,00012 | CPZ-C3 | 0,176 | 0,187 | 7 | 0,00742 |
| CP3-F1 | 0,143 | 0,158 | 12 | 0,00057 | CPZ-CZ | 0,241 | 0,257 | 8 | 0,00117 |
| CP3-C6 | 0,151 | 0,167 | 12 | 0,00084 | CPZ-P1 | 0,211 | 0,224 | 7 | 0,00526 |
| CPZ-C4 | 0,196 | 0,219 | 12 | 0,00023 | P1-CPZ | 0,211 | 0,224 | 7 | 0,00526 |
| CPZ-CP1 | 0,225 | 0,249 | 12 | 0,00039 | POZ-F1 | 0,082 | 0,0888 | 10 | 0,01542 |
| CPZ-C6 | 0,166 | 0,185 | 13 | 0,00012 |  |  |  |  |  |
| TP8-C5 | 0,142 | 0,157 | 12 | 0,00253 |  |  |  |  |  |
| P1-PZ | 0,227 | 0,252 | 12 | 0,00090 |  |  |  |  |  |
| P2-FC1 | 0,182 | 0,205 | 14 | 0,00078 |  |  |  |  |  |
| P2-C5 | 0,154 | 0,17 | 12 | 0,00177 |  |  |  |  |  |
| PO7-P3 | 0,187 | 0,214 | 16 | 0,00004 |  |  |  |  |  |
| PO7-F6 | 0,165 | 0,186 | 14 | 0,00025 |  |  |  |  |  |
| PO8-O1 | 0,172 | 0,197 | 16 | 0,00002 |  |  |  |  |  |
| PO8-CP2 | 0,172 | 0,191 | 12 | 0,00023 |  |  |  |  |  |
| PO8-C5 | 0,147 | 0,163 | 12 | 0,00041 |  |  |  |  |  |

Table 4-S – Synchronization Entropy: the most significant differences between Pre and Post conditions in Mograine patients; results by ANOVA test with the Bonferroni-Holmes correction for alpha band and beta bands are shown.
