# Supplementary material for: Functional Connectivity of EEG Signals Under Laser Stimulation in Migraine
Source: Front Hum Neurosci. 2015 Nov 24;9:640. doi: 10.3389/fnhum.2015.00640 (PMC4656845; doi:10.3389/fnhum.2015.00640)
Supplement: Supplementary file 5 [file Table_5.DOCX]

|  | | | | | | |  |  |  |
| --- | --- | --- | --- | --- | --- | --- | --- | --- | --- |
| delta |  |  |  |  | theta |  |  |  |  |
| Couples | MIGR (bits) | CONT (bits) | Percentual  difference | T-Test  p-value | Couples | MIGR (bits) | CONT (bits) | Percentual difference | T-Test p-value |
| FPZ-AF4 | 0,202 | 0,202 | 18 | 3,05E-06 | FPZ-AF4 | 0,136 | 0,171 | 22 | 1,17E-09 |
| F8-FC2 | 0,181 | 0,181 | 20 | 7,49E-07 | F7-FC4 | 0,12 | 0,151 | 21 | 2,81E-09 |
| T3-C3 | 0,22 | 0,22 | 19 | 1,53E-06 | F4-FC2 | 0,156 | 0,199 | 22 | 9,14E-09 |
| T3-CP1 | 0,206 | 0,206 | 19 | 1,76E-06 | F4-FC5 | 0,132 | 0,168 | 22 | 1,42E-09 |
| T3-CPZ | 0,209 | 0,209 | 20 | 4,52E-07 | F4-FT7 | 0,113 | 0,144 | 23 | 4,57E-07 |
| C3-T3 | 0,22 | 0,22 | 19 | 1,53E-06 | T3-PZ | 0,144 | 0,184 | 23 | 4,44E-11 |
| CZ-CP5 | 0,217 | 0,217 | 18 | 3,36E-06 | T3-FC1 | 0,148 | 0,187 | 22 | 5,55E-09 |
| C4-PO7 | 0,198 | 0,198 | 18 | 1,94E-06 | T3-CP1 | 0,139 | 0,178 | 23 | 6,12E-11 |
| P3-TP8 | 0,136 | 0,136 | 33 | 1,61E-07 | T3-FT8 | 0,139 | 0,174 | 21 | 7,46E-07 |
| P4-TP8 | 0,156 | 0,156 | 34 | 1,08E-07 | T3-CPZ | 0,14 | 0,185 | 25 | 1,15E-12 |
| OZ-FT7 | 0,187 | 0,187 | 24 | 2,41E-07 | T5-FC1 | 0,148 | 0,186 | 21 | 7,24E-09 |
| OZ-PO7 | 0,23 | 0,23 | 23 | 6,01E-08 | P3-CP3 | 0,241 | 0,19 | 28 | 1,31E-07 |
| F6-TP8 | 0,147 | 0,147 | 36 | 1,87E-08 | P3-TP8 | 0,162 | 0,124 | 32 | 7,82E-08 |
| FC2-F8 | 0,181 | 0,181 | 20 | 7,49E-07 | PZ-T3 | 0,144 | 0,184 | 23 | 4,44E-11 |
| FC2-CP5 | 0,199 | 0,199 | 20 | 2,95E-06 | PZ-FT7 | 0,133 | 0,169 | 22 | 2,35E-08 |
| CP1-T3 | 0,206 | 0,206 | 19 | 1,76E-06 | F6-TP8 | 0,187 | 0,136 | 38 | 4,43E-10 |
| CP1-FT7 | 0,197 | 0,197 | 21 | 9,42E-07 | FC2-F4 | 0,156 | 0,199 | 22 | 9,14E-09 |
| PO3-PO7 | 0,219 | 0,219 | 21 | 2,54E-07 | FC2-FC5 | 0,158 | 0,2 | 22 | 3,07E-09 |
| CP5-CZ | 0,217 | 0,217 | 18 | 3,36E-06 | FC1-T3 | 0,148 | 0,187 | 22 | 5,55E-09 |
| CP5-FC2 | 0,199 | 0,199 | 20 | 2,95E-06 | FC1-T5 | 0,148 | 0,186 | 21 | 7,24E-09 |
| AF4-FPZ | 0,202 | 0,202 | 18 | 3,05E-06 | FC1-FT7 | 0,137 | 0,171 | 21 | 9,47E-07 |
| FT7-OZ | 0,187 | 0,187 | 24 | 2,41E-07 | CP1-T3 | 0,139 | 0,178 | 23 | 6,12E-11 |
| FT7-CP1 | 0,197 | 0,197 | 21 | 9,42E-07 | CP1-FT7 | 0,13 | 0,163 | 21 | 3,35E-07 |
| FT7-PO7 | 0,186 | 0,186 | 20 | 2,94E-06 | FC5-F4 | 0,132 | 0,168 | 22 | 1,42E-09 |
| CPZ-T3 | 0,209 | 0,209 | 20 | 4,52E-07 | FC5-FC2 | 0,158 | 0,2 | 22 | 3,07E-09 |
| TP8-P3 | 0,136 | 0,136 | 33 | 1,61E-07 | CP5-FT7 | 0,129 | 0,163 | 22 | 1,69E-06 |
| TP8-P4 | 0,156 | 0,156 | 34 | 1,08E-07 | AF4-FPZ | 0,136 | 0,171 | 22 | 1,17E-09 |
| TP8-F6 | 0,147 | 0,147 | 36 | 1,87E-08 | FT7-F4 | 0,113 | 0,144 | 23 | 4,57E-07 |
| TP8-PO8 | 0,16 | 0,16 | 30 | 2,29E-06 | FT7-PZ | 0,133 | 0,169 | 22 | 2,35E-08 |
| PO7-C4 | 0,198 | 0,198 | 18 | 1,94E-06 | FT7-FC1 | 0,137 | 0,171 | 21 | 9,47E-07 |
| PO7-OZ | 0,23 | 0,23 | 23 | 6,01E-08 | FT7-CP1 | 0,13 | 0,163 | 21 | 3,35E-07 |
| PO7-PO3 | 0,219 | 0,219 | 21 | 2,54E-07 | FT7-CP5 | 0,129 | 0,163 | 22 | 1,69E-06 |
| PO7-FT7 | 0,186 | 0,186 | 20 | 2,94E-06 | FT7-C6 | 0,13 | 0,167 | 23 | 1,98E-07 |
| PO7-POZ | 0,232 | 0,232 | 19 | 2,31E-06 | FT7-CPZ | 0,134 | 0,168 | 21 | 1,75E-07 |
| POZ-PO7 | 0,232 | 0,232 | 19 | 2,31E-06 | FCZ-C1 | 0,237 | 0,195 | 23 | 1,52E-06 |
| PO8-TP8 | 0,16 | 0,16 | 30 | 2,29E-06 | FC4-F7 | 0,12 | 0,151 | 21 | 2,81E-09 |
|  |  |  |  |  | FT8-T3 | 0,139 | 0,174 | 21 | 7,46E-07 |
|  |  |  |  |  | C1-FCZ | 0,237 | 0,195 | 23 | 1,52E-06 |
|  |  |  |  |  | C6-FT7 | 0,13 | 0,167 | 23 | 1,98E-07 |
|  |  |  |  |  | CP3-P3 | 0,241 | 0,19 | 28 | 1,31E-07 |
|  |  |  |  |  | CP3-TP8 | 0,158 | 0,117 | 36 | 4,20E-09 |
|  |  |  |  |  | CPZ-T3 | 0,14 | 0,185 | 25 | 1,15E-12 |
|  |  |  |  |  | CPZ-FT7 | 0,134 | 0,168 | 21 | 1,75E-07 |
|  |  |  |  |  | TP8-P3 | 0,162 | 0,124 | 32 | 7,82E-08 |
|  |  |  |  |  | TP8-F6 | 0,187 | 0,136 | 38 | 4,43E-10 |
|  |  |  |  |  | TP8-CP3 | 0,158 | 0,117 | 36 | 4,20E-09 |
|  |  |  |  |  | TP8-P2 | 0,177 | 0,134 | 33 | 6,79E-07 |
|  |  |  |  |  | P2-TP8 | 0,177 | 0,134 | 33 | 6,79E-07 |

Table 5-S – Synchronization Entropy (SE) for delta and theta bands : the most significant differences between MIGR (migraine patients) and CONT (controls) are reported ; blue colors express a reduction and red colors an increase of SE in MIGR vs CONT
