# Supplementary material for: Functional Connectivity of EEG Signals Under Laser Stimulation in Migraine
Source: Front Hum Neurosci. 2015 Nov 24;9:640. doi: 10.3389/fnhum.2015.00640 (PMC4656845; doi:10.3389/fnhum.2015.00640)
Supplement: Supplementary file 6 [file Table_6.DOCX]

| alpha |  |  |  |  | beta |  |  |  |  |
| --- | --- | --- | --- | --- | --- | --- | --- | --- | --- |
| Couples | MIGR (bits) | CONT (bits) | Percentual difference | T-Test p-value | Couples | MIGR (bits) | CONT (bits) | Percentual difference | T-Test p-value |
| F7-PO4 | 0,0861 | 0,123 | 31 | 8,15E-13 | F7-PO4 | 0,0377 | 0,0736 | 50 | 1,94E-18 |
| F7-FT8 | 0,0901 | 0,13 | 31 | 1,55E-15 | F4-FC2 | 0,0683 | 0,13 | 49 | 1,20E-24 |
| T3-C3 | 0,128 | 0,185 | 32 | 1,56E-24 | F4-FC3 | 0,0579 | 0,11 | 48 | 6,85E-34 |
| T3-CZ | 0,0964 | 0,134 | 29 | 1,59E-18 | F8-FT7 | 0,0341 | 0,0648 | 48 | 2,45E-18 |
| T3-PZ | 0,109 | 0,152 | 29 | 7,04E-20 | T3-C3 | 0,0628 | 0,117 | 47 | 1,68E-35 |
| T3-FC2 | 0,0922 | 0,128 | 29 | 7,32E-15 | T3-CZ | 0,044 | 0,081 | 47 | 1,01E-36 |
| T3-CP1 | 0,0983 | 0,141 | 31 | 1,41E-20 | T3-FC2 | 0,0427 | 0,0763 | 45 | 6,17E-30 |
| T3-PO4 | 0,0879 | 0,123 | 29 | 7,45E-13 | T3-FT8 | 0,0373 | 0,0722 | 49 | 3,09E-19 |
| T3-FT8 | 0,098 | 0,141 | 31 | 1,21E-13 | T3-C5 | 0,0816 | 0,149 | 46 | 3,00E-29 |
| T3-C2 | 0,0896 | 0,13 | 32 | 4,92E-24 | T3-TP7 | 0,115 | 0,209 | 46 | 9,75E-40 |
| T3-CPZ | 0,106 | 0,146 | 29 | 4,77E-19 | T3-CPZ | 0,047 | 0,085 | 46 | 6,86E-37 |
| C3-T3 | 0,128 | 0,185 | 32 | 1,56E-24 | C3-T3 | 0,0628 | 0,117 | 47 | 1,68E-35 |
| CZ-T3 | 0,0964 | 0,134 | 29 | 1,59E-18 | CZ-T3 | 0,044 | 0,081 | 47 | 1,01E-36 |
| C4-FT7 | 0,0908 | 0,126 | 29 | 3,52E-13 | C4-T4 | 0,063 | 0,119 | 48 | 1,13E-35 |
| T4-FC5 | 0,0983 | 0,138 | 30 | 2,08E-16 | C4-P2 | 0,169 | 0,14 | 21 | 1,88E-06 |
| T4-FT7 | 0,0928 | 0,136 | 33 | 2,64E-14 | T4-C4 | 0,063 | 0,119 | 48 | 1,13E-35 |
| P3-CP3 | 0,252 | 0,206 | 23 | 1,60E-06 | T4-FC5 | 0,0385 | 0,0743 | 49 | 1,49E-22 |
| P3-TP8 | 0,129 | 0,103 | 26 | 2,39E-06 | T4-CP5 | 0,0889 | 0,174 | 50 | 1,68E-42 |
| PZ-T3 | 0,109 | 0,152 | 29 | 7,04E-20 | T4-FT7 | 0,0351 | 0,0659 | 48 | 1,33E-18 |
| P4-TP8 | 0,192 | 0,146 | 33 | 1,28E-08 | T4-C2 | 0,0529 | 0,0964 | 46 | 8,37E-33 |
| T6-F6 | 0,2 | 0,161 | 25 | 9,36E-10 | T4-C6 | 0,0796 | 0,162 | 52 | 1,69E-41 |
| O1-TP8 | 0,14 | 0,109 | 29 | 2,40E-07 | P3-CP3 | 0,266 | 0,205 | 31 | 2,45E-13 |
| OZ-TP8 | 0,153 | 0,112 | 37 | 5,71E-10 | P4-TP8 | 0,14 | 0,103 | 37 | 5,18E-08 |
| F6-T6 | 0,2 | 0,161 | 25 | 9,36E-10 | T6-F6 | 0,142 | 0,0989 | 45 | 8,18E-16 |
| F6-CP4 | 0,178 | 0,136 | 32 | 9,76E-10 | T6-TP8 | 0,18 | 0,112 | 62 | 2,44E-15 |
| F6-TP8 | 0,186 | 0,107 | 75 | 1,62E-27 | O1-TP8 | 0,0754 | 0,0567 | 34 | 2,94E-07 |
| FC2-T3 | 0,0922 | 0,128 | 29 | 7,32E-15 | OZ-TP8 | 0,096 | 0,0585 | 65 | 6,74E-17 |
| FC2-TP7 | 0,0975 | 0,136 | 29 | 3,73E-15 | F6-T6 | 0,142 | 0,0989 | 45 | 8,18E-16 |
| CP1-T3 | 0,0983 | 0,141 | 31 | 1,41E-20 | F6-CP4 | 0,135 | 0,103 | 32 | 1,17E-07 |
| PO3-TP8 | 0,174 | 0,137 | 28 | 7,11E-07 | F6-TP8 | 0,125 | 0,0618 | 103 | 1,17E-25 |
| PO4-F7 | 0,0861 | 0,123 | 31 | 8,15E-13 | F6-P6 | 0,176 | 0,142 | 25 | 1,57E-06 |
| PO4-T3 | 0,0879 | 0,123 | 29 | 7,45E-13 | F6-PO8 | 0,252 | 0,181 | 40 | 5,43E-21 |
| FC5-T4 | 0,0983 | 0,138 | 30 | 2,08E-16 | FC2-F4 | 0,0683 | 0,13 | 49 | 1,20E-24 |
| FC5-C6 | 0,106 | 0,148 | 30 | 1,17E-16 | FC2-T3 | 0,0427 | 0,0763 | 45 | 6,17E-30 |
| FT7-C4 | 0,0908 | 0,126 | 29 | 3,52E-13 | PO3-TP8 | 0,121 | 0,0828 | 47 | 7,28E-11 |
| FT7-T4 | 0,0928 | 0,136 | 33 | 2,64E-14 | PO4-F7 | 0,0377 | 0,0736 | 50 | 1,94E-18 |
| FT7-C2 | 0,0866 | 0,125 | 32 | 1,63E-15 | FC5-T4 | 0,0385 | 0,0743 | 49 | 1,49E-22 |
| FT7-C6 | 0,0917 | 0,13 | 30 | 1,70E-13 | FC5-C6 | 0,0482 | 0,0944 | 50 | 2,53E-25 |
| FT8-F7 | 0,0901 | 0,13 | 31 | 1,55E-15 | CP5-T4 | 0,0889 | 0,174 | 50 | 1,68E-42 |
| FT8-T3 | 0,098 | 0,141 | 31 | 1,21E-13 | FT7-F8 | 0,0341 | 0,0648 | 48 | 2,45E-18 |
| C2-T3 | 0,0896 | 0,13 | 32 | 4,92E-24 | FT7-T4 | 0,0351 | 0,0659 | 48 | 1,33E-18 |
| C2-FT7 | 0,0866 | 0,125 | 32 | 1,63E-15 | FC3-F4 | 0,0579 | 0,11 | 48 | 6,85E-34 |
| C6-FC5 | 0,106 | 0,148 | 30 | 1,17E-16 | FT8-T3 | 0,0373 | 0,0722 | 49 | 3,09E-19 |
| C6-FT7 | 0,0917 | 0,13 | 30 | 1,70E-13 | C5-T3 | 0,0816 | 0,149 | 46 | 3,00E-29 |
| TP7-FC2 | 0,0975 | 0,136 | 29 | 3,73E-15 | C2-T4 | 0,0529 | 0,0964 | 46 | 8,37E-33 |
| CP3-P3 | 0,252 | 0,206 | 23 | 1,60E-06 | C6-T4 | 0,0796 | 0,162 | 52 | 1,69E-41 |
| CPZ-T3 | 0,106 | 0,146 | 29 | 4,77E-19 | C6-FC5 | 0,0482 | 0,0944 | 50 | 2,53E-25 |
| CP4-F6 | 0,178 | 0,136 | 32 | 9,76E-10 | TP7-T3 | 0,115 | 0,209 | 46 | 9,75E-40 |
| TP8-P3 | 0,129 | 0,103 | 26 | 2,39E-06 | CP3-P3 | 0,266 | 0,205 | 31 | 2,45E-13 |
| TP8-P4 | 0,192 | 0,146 | 33 | 1,28E-08 | CP3-P1 | 0,233 | 0,186 | 26 | 2,92E-12 |
| TP8-O1 | 0,14 | 0,109 | 29 | 2,40E-07 | CPZ-T3 | 0,047 | 0,085 | 46 | 6,86E-37 |
| TP8-OZ | 0,153 | 0,112 | 37 | 5,71E-10 | CP4-F6 | 0,135 | 0,103 | 32 | 1,17E-07 |
| TP8-F6 | 0,186 | 0,107 | 75 | 1,62E-27 | CP4-P2 | 0,224 | 0,187 | 21 | 2,41E-06 |
| TP8-PO3 | 0,174 | 0,137 | 28 | 7,11E-07 | CP4-POZ | 0,151 | 0,12 | 26 | 2,50E-08 |
| TP8-P2 | 0,163 | 0,123 | 33 | 1,79E-08 | TP8-P4 | 0,14 | 0,103 | 37 | 5,18E-08 |
| TP8-POZ | 0,152 | 0,122 | 26 | 5,09E-07 | TP8-T6 | 0,18 | 0,112 | 62 | 2,44E-15 |
| TP8-PO8 | 0,197 | 0,142 | 40 | 9,46E-13 | TP8-O1 | 0,0754 | 0,0567 | 34 | 2,94E-07 |
| P2-TP8 | 0,163 | 0,123 | 33 | 1,79E-08 | TP8-OZ | 0,096 | 0,0585 | 65 | 6,74E-17 |
| POZ-TP8 | 0,152 | 0,122 | 26 | 5,09E-07 | TP8-F6 | 0,125 | 0,0618 | 103 | 1,17E-25 |
| PO8-TP8 | 0,197 | 0,142 | 40 | 9,46E-13 | TP8-PO3 | 0,121 | 0,0828 | 47 | 7,28E-11 |
|  |  |  |  |  | TP8-P2 | 0,113 | 0,0756 | 50 | 4,97E-12 |
|  |  |  |  |  | TP8-P6 | 0,183 | 0,138 | 33 | 1,57E-07 |
|  |  |  |  |  | TP8-POZ | 0,0924 | 0,066 | 41 | 1,81E-10 |
|  |  |  |  |  | TP8-PO8 | 0,148 | 0,094 | 58 | 2,96E-16 |
|  |  |  |  |  | P1-CP3 | 0,233 | 0,186 | 26 | 2,92E-12 |
|  |  |  |  |  | P2-C4 | 0,169 | 0,14 | 21 | 1,88E-06 |
|  |  |  |  |  | P2-CP4 | 0,224 | 0,187 | 21 | 2,41E-06 |
|  |  |  |  |  | P2-TP8 | 0,113 | 0,0756 | 50 | 4,97E-12 |
|  |  |  |  |  | P6-F6 | 0,176 | 0,142 | 25 | 1,57E-06 |
|  |  |  |  |  | P6-TP8 | 0,183 | 0,138 | 33 | 1,57E-07 |
|  |  |  |  |  | POZ-CP4 | 0,151 | 0,12 | 26 | 2,50E-08 |
|  |  |  |  |  | POZ-TP8 | 0,0924 | 0,066 | 41 | 1,81E-10 |
|  |  |  |  |  | PO8-F6 | 0,252 | 0,181 | 40 | 5,43E-21 |
|  |  |  |  |  | PO8-TP8 | 0,148 | 0,094 | 58 | 2,96E-16 |

Table 6-S – Synchronization Entropy (SE) for alpha and beta bands : the most significant differences between MIGR (migraine patients) and CONT (controls) are reported ; blue colors express a reduction and red colors an increase of SE in MIGR vs CONT
