# Supplementary material for: Functional Connectivity of EEG Signals Under Laser Stimulation in Migraine
Source: Front Hum Neurosci. 2015 Nov 24;9:640. doi: 10.3389/fnhum.2015.00640 (PMC4656845; doi:10.3389/fnhum.2015.00640)
Supplement: Supplementary file 7 [file Table_7.DOCX]

| delta |  |  |  |  | theta |  |  |  |  |
| --- | --- | --- | --- | --- | --- | --- | --- | --- | --- |
| Couples | MIGR (bits) | CONT (bits) | Percentual  difference | T-Test  p-value | Couples | MIGR (bits) | CONT (bits) | Percentual difference | T-Test p-value |
| F3-TP8 | 1,06 | 0,786 | 36 | 4,60E-13 | F3-TP8 | 1,34 | 0,973 | 38 | 4,07E-15 |
| P3-TP8 | 0,983 | 0,708 | 40 | 6,83E-16 | C3-TP8 | 1,27 | 0,928 | 37 | 2,73E-18 |
| P4-TP8 | 0,981 | 0,706 | 40 | 3,25E-16 | T5-TP8 | 1,29 | 0,951 | 37 | 8,27E-18 |
| F6-TP8 | 0,979 | 0,706 | 40 | 2,73E-15 | P3-TP8 | 1,26 | 0,829 | 53 | 1,39E-26 |
| FC6-TP8 | 1,01 | 0,757 | 35 | 8,73E-15 | P3-P2 | 1,26 | 0,909 | 40 | 9,30E-21 |
| C1-P2 | 0,967 | 0,73 | 34 | 1,09E-13 | P4-TP8 | 1,24 | 0,856 | 46 | 1,18E-21 |
| CP3-TP8 | 0,988 | 0,652 | 52 | 4,16E-23 | P4-P2 | 1,24 | 0,906 | 38 | 1,96E-19 |
| CP3-P2 | 0,971 | 0,703 | 39 | 5,28E-17 | F6-TP8 | 1,21 | 0,832 | 46 | 6,58E-21 |
| TP8-FPZ | 0,938 | 0,703 | 34 | 7,90E-14 | CP1-TP8 | 1,24 | 0,898 | 39 | 1,15E-18 |
| TP8-FP2 | 0,931 | 0,691 | 36 | 3,16E-12 | CP2-TP8 | 1,22 | 0,893 | 38 | 6,86E-18 |
| TP8-F3 | 0,983 | 0,719 | 38 | 3,49E-13 | FC6-TP8 | 1,3 | 0,932 | 41 | 1,37E-20 |
| TP8-FZ | 1,03 | 0,771 | 34 | 3,89E-14 | FC3-TP8 | 1,31 | 0,959 | 38 | 1,20E-18 |
| TP8-CZ | 1,04 | 0,785 | 34 | 3,33E-14 | C5-TP8 | 1,31 | 0,949 | 39 | 4,51E-20 |
| TP8-C4 | 1,02 | 0,769 | 34 | 3,96E-14 | C1-TP8 | 1,22 | 0,894 | 38 | 9,54E-18 |
| TP8-T4 | 0,995 | 0,742 | 35 | 9,31E-15 | C1-P2 | 1,22 | 0,898 | 37 | 6,12E-18 |
| TP8-P3 | 1,03 | 0,734 | 41 | 3,51E-17 | C2-TP8 | 1,23 | 0,899 | 38 | 3,23E-18 |
| TP8-P4 | 1 | 0,718 | 41 | 5,80E-17 | CP3-P4 | 1,29 | 0,953 | 37 | 6,79E-25 |
| TP8-F6 | 1,02 | 0,719 | 42 | 2,25E-17 | CP3-FCZ | 1,3 | 0,946 | 39 | 4,16E-27 |
| TP8-AFZ | 1 | 0,755 | 34 | 5,69E-14 | CP3-C1 | 1,28 | 0,937 | 38 | 9,31E-25 |
| TP8-AF7 | 0,998 | 0,741 | 36 | 3,15E-15 | CP3-TP8 | 1,26 | 0,785 | 61 | 6,77E-31 |
| TP8-AF4 | 0,997 | 0,743 | 35 | 5,46E-15 | CP3-P2 | 1,25 | 0,869 | 45 | 1,78E-23 |
| TP8-F1 | 1,03 | 0,771 | 34 | 1,97E-14 | CP3-PO8 | 1,3 | 0,946 | 39 | 1,43E-26 |
| TP8-F2 | 1 | 0,755 | 34 | 9,43E-14 | TP8-FP2 | 1,21 | 0,882 | 38 | 9,39E-15 |
| TP8-FT7 | 0,945 | 0,706 | 35 | 8,69E-10 | TP8-F3 | 1,26 | 0,908 | 40 | 5,04E-15 |
| TP8-FT8 | 1 | 0,755 | 34 | 3,93E-14 | TP8-P3 | 1,31 | 0,925 | 42 | 2,18E-19 |
| TP8-C1 | 1,04 | 0,783 | 34 | 3,27E-14 | TP8-P4 | 1,28 | 0,894 | 44 | 1,12E-20 |
| TP8-C2 | 1,03 | 0,778 | 34 | 3,11E-14 | TP8-F6 | 1,29 | 0,901 | 44 | 3,56E-20 |
| TP8-C6 | 0,997 | 0,747 | 34 | 2,78E-14 | TP8-CP3 | 1,31 | 0,865 | 52 | 2,61E-25 |
| TP8-CP3 | 1,03 | 0,682 | 53 | 1,33E-23 | TP8-P2 | 1,26 | 0,895 | 42 | 2,40E-16 |
| TP8-P2 | 0,992 | 0,714 | 40 | 4,45E-14 | TP8-PO8 | 1,29 | 0,892 | 45 | 5,33E-21 |
| TP8-PO8 | 1,01 | 0,711 | 43 | 1,48E-17 | P2-P4 | 1,2 | 0,872 | 38 | 2,64E-19 |
| P2-C1 | 0,981 | 0,739 | 34 | 7,09E-14 | P2-C1 | 1,24 | 0,909 | 38 | 5,30E-18 |
| P2-CP3 | 0,971 | 0,705 | 39 | 8,64E-17 | P2-CP3 | 1,23 | 0,877 | 41 | 2,58E-20 |
| P2-TP8 | 0,942 | 0,69 | 37 | 1,64E-12 | P2-TP8 | 1,18 | 0,828 | 44 | 2,51E-17 |
| P2-PO8 | 0,974 | 0,724 | 36 | 2,73E-15 | P2-PO8 | 1,23 | 0,9 | 37 | 4,61E-18 |
| PO8-TP8 | 0,977 | 0,692 | 42 | 1,26E-17 | PO8-TP8 | 1,24 | 0,845 | 48 | 2,07E-22 |
| PO8-P2 | 1,01 | 0,74 | 37 | 8,59E-17 | PO8-P2 | 1,27 | 0,932 | 37 | 2,80E-18 |

Table 7-S – Granger Causality (GC) for delta and theta bands : the most significant differences between MIGR (migraine patients) and CONT (controls) are reported ; blue colors express a reduction and red colors an increase of GC in MIGR vs CONT.
