# Supplementary material for: Functional Connectivity of EEG Signals Under Laser Stimulation in Migraine
Source: Front Hum Neurosci. 2015 Nov 24;9:640. doi: 10.3389/fnhum.2015.00640 (PMC4656845; doi:10.3389/fnhum.2015.00640)
Supplement: Supplementary file 8 [file Table_8.DOCX]

| alpha |  |  |  |  | beta |  |  |  |  |
| --- | --- | --- | --- | --- | --- | --- | --- | --- | --- |
| Couples | MIGR (bits) | CONT (bits) | Percentual difference | T-Test  p-value | Couples | MIGR (bits) | CONT (bits) | Percentual difference | T-Test  p-value |
| FP2-TP8 | 1,77 | 1,34 | 33 | 2,84E-14 | FP2-TP8 | 1,61 | 1,12 | 45 | 6,31E-16 |
| F3-TP8 | 1,55 | 1,18 | 33 | 7,60E-13 | P3-TP8 | 1,3 | 0,876 | 50 | 7,65E-27 |
| F8-TP8 | 1,62 | 1,24 | 32 | 4,50E-16 | P4-TP8 | 1,27 | 0,907 | 41 | 3,86E-20 |
| CZ-TP8 | 1,43 | 1,08 | 33 | 3,38E-15 | T6-TP8 | 1,22 | 0,871 | 42 | 1,10E-22 |
| P3-TP8 | 1,35 | 0,96 | 42 | 1,20E-17 | F6-TP8 | 1,21 | 0,838 | 45 | 9,95E-22 |
| P3-P2 | 1,37 | 1,05 | 31 | 1,02E-13 | CP3-TP8 | 1,34 | 0,883 | 52 | 5,66E-29 |
| P4-TP8 | 1,32 | 0,982 | 35 | 1,19E-13 | TP8-FP1 | 1,15 | 0,833 | 39 | 1,22E-13 |
| F6-TP8 | 1,26 | 0,953 | 33 | 6,59E-11 | TP8-FP2 | 1,2 | 0,835 | 44 | 4,77E-19 |
| FC1-TP8 | 1,35 | 1,03 | 33 | 7,32E-13 | TP8-F3 | 1,2 | 0,828 | 46 | 8,93E-20 |
| FC5-TP8 | 1,53 | 1,18 | 31 | 2,59E-15 | TP8-F8 | 1,23 | 0,896 | 39 | 1,67E-20 |
| FC6-TP8 | 1,42 | 1,07 | 33 | 3,57E-15 | TP8-CZ | 1,23 | 0,895 | 39 | 9,92E-21 |
| FCZ-P2 | 1,49 | 1,14 | 32 | 8,03E-16 | TP8-C4 | 1,24 | 0,896 | 39 | 1,70E-20 |
| C5-TP8 | 1,45 | 1,11 | 32 | 2,77E-15 | TP8-T4 | 1,24 | 0,892 | 40 | 2,12E-21 |
| C1-CP3 | 1,47 | 1,11 | 34 | 1,84E-22 | TP8-P3 | 1,24 | 0,84 | 49 | 4,92E-25 |
| C1-TP8 | 1,43 | 1,09 | 33 | 2,74E-15 | TP8-P4 | 1,23 | 0,817 | 52 | 3,97E-28 |
| C1-P2 | 1,44 | 1,07 | 36 | 6,05E-18 | TP8-F6 | 1,24 | 0,804 | 55 | 1,99E-29 |
| C2-TP8 | 1,41 | 1,09 | 31 | 4,50E-14 | TP8-CP1 | 1,23 | 0,896 | 39 | 1,68E-20 |
| CP3-P4 | 1,44 | 1,1 | 31 | 5,33E-20 | TP8-FC5 | 1,24 | 0,9 | 39 | 1,95E-20 |
| CP3-F6 | 1,45 | 1,09 | 33 | 3,71E-21 | TP8-FC6 | 1,24 | 0,899 | 39 | 1,28E-20 |
| CP3-FCZ | 1,43 | 1,09 | 33 | 1,70E-21 | TP8-CP6 | 1,24 | 0,897 | 39 | 1,15E-20 |
| CP3-C1 | 1,42 | 1,06 | 35 | 1,48E-22 | TP8-AF7 | 1,23 | 0,893 | 39 | 9,32E-21 |
| CP3-TP8 | 1,39 | 0,906 | 54 | 3,86E-26 | TP8-AF4 | 1,24 | 0,898 | 39 | 1,34E-20 |
| CP3-P5 | 1,39 | 1,07 | 31 | 8,96E-17 | TP8-F2 | 1,24 | 0,898 | 39 | 1,33E-20 |
| CP3-P2 | 1,39 | 0,987 | 42 | 1,49E-21 | TP8-FT7 | 1,15 | 0,817 | 42 | 6,42E-14 |
| CP3-PO8 | 1,45 | 1,09 | 34 | 8,19E-22 | TP8-FC3 | 1,24 | 0,898 | 39 | 1,38E-20 |
| CPZ-TP8 | 1,37 | 1,05 | 31 | 1,59E-13 | TP8-FC4 | 1,24 | 0,895 | 39 | 8,59E-21 |
| TP8-FP2 | 1,31 | 1 | 32 | 2,95E-11 | TP8-FT8 | 1,24 | 0,895 | 39 | 4,09E-21 |
| TP8-F3 | 1,34 | 1,03 | 31 | 2,98E-10 | TP8-C5 | 1,24 | 0,899 | 39 | 1,85E-20 |
| TP8-P3 | 1,39 | 1,05 | 34 | 1,42E-13 | TP8-C2 | 1,24 | 0,897 | 39 | 1,88E-20 |
| TP8-P4 | 1,38 | 1,01 | 37 | 1,30E-15 | TP8-C6 | 1,23 | 0,893 | 39 | 5,33E-21 |
| TP8-F6 | 1,38 | 1,02 | 36 | 1,21E-14 | TP8-TP7 | 1,24 | 0,899 | 39 | 1,28E-20 |
| TP8-CP3 | 1,39 | 0,98 | 43 | 1,39E-18 | TP8-CP3 | 1,24 | 0,829 | 50 | 1,97E-25 |
| TP8-P2 | 1,36 | 1,02 | 35 | 1,60E-12 | TP8-CPZ | 1,24 | 0,897 | 39 | 1,39E-20 |
| TP8-PO8 | 1,38 | 1,02 | 37 | 5,03E-15 | TP8-P2 | 1,2 | 0,804 | 50 | 5,17E-22 |
| P2-CP3 | 1,33 | 1,01 | 33 | 4,72E-14 | TP8-PO8 | 1,24 | 0,813 | 53 | 2,63E-28 |
| P2-TP8 | 1,28 | 0,965 | 33 | 5,85E-11 | P2-TP8 | 1,26 | 0,901 | 40 | 6,74E-17 |
| PO8-TP8 | 1,29 | 0,956 | 36 | 3,12E-13 | PO8-TP8 | 1,21 | 0,827 | 48 | 1,63E-24 |

Table 8-S – Granger Causality (GC) for alpha and beta bands : the most significant differences between MIGR (migraine patients) and CONT (controls) are reported ; blue colors express a reduction and red colors an increase of GC in MIGR vs CONT.
