# Supplementary material for: Functional Connectivity of EEG Signals Under Laser Stimulation in Migraine
Source: Front Hum Neurosci. 2015 Nov 24;9:640. doi: 10.3389/fnhum.2015.00640 (PMC4656845; doi:10.3389/fnhum.2015.00640)
Supplement: Supplementary file 9 [file Data_Sheet_1.DOCX]

**Supplementary material**

**Functional connectivity of EEG signals under laser stimulation in migraine**

Marina de Tommaso+°, Gabriele Trotta*, Eleonora Vecchio°, Katia Ricci°, Frederik Van de Steen§, Anna Montemurno°, Marta Lorenzo°, Daniele Marinazzo§, Roberto Bellotti* and Sebastiano Stramaglia*

° Basic Medical Neuroscience and Sensory System Department, Bari Aldo Moro University

*Physic Department, Bari Aldo Moro University

° * TIRES Center, Bari Aldo Moro University

§ Department of Data Analysis Faculty of Psychological and Pedagogical Sciences 1, Gent University

Corresponding author+

Prof. Marina de Tommaso

Basic Medical Neuroscience and Sensory System Department, Bari Aldo Moro University

Policlinico General Hospital

Via Amendola 207 A 70124 Bari

e-mail: [marina.detommaso@uniba.it](mailto:marina.detommaso@uniba.it)

Tel 00390805596859 Fax 00390805478532

In order to introduce nonlinear Granger Causality, we first review the linear case briefly. The temporal dynamics of a stationary time series$\{x(t)\}_{t=1,.,N+m}$, can be described using an autoregressive model based on the past *m* values of the time series,

$x_{n}=\sum_{j=1}^{m} \mathbf{a}_{j}x_{n-j}+\mathbf{e}_{n}$;

in order to include information from a simultaneously recorded time series $\{y(t)\}_{t=1,.,N+m}$, we could consider instead a bivariate autoregressive model which also takes into account the past values of $y$:

.

The coefficients of the models are estimated using a standard least squares optimization; $m$ is the order of the autoregressive model and is usually chosen according to Schwartz criterion (1978) that we used in this work, or other order selection criteria. The concept of Granger causality is that $y$ Granger-causes $x$ if the variance of residuals $\mathbf{e}'$ is significantly smaller than the variance of residuals$\mathbf{e}$, as happens when the coefficients $\mathbf{b}$ are all significantly different from zero. An index measuring the strength of the causal interaction is then defined as

, (1)

where denotes averaging over $n$ (note that $\left\langle\mathbf{e}\right\rangle=\left\langle\mathbf{e}'\right\rangle\langle\rangle=0$). Exchanging the roles of the two series, we could evaluate the causality index in the opposite direction ($x\to y$).

In () it has been shown that one may write

$\delta=\sum_{i=1}^{m} r_{i}^{2}$, (2)

where $r_{i}$ are suitable Pearson correlation coefficients. Evaluating the probability $p_{i}$ that $r_{i}$ is due to chance, under the null hypothesis of no correlation, we can avoid false causalities and compensate the threshold of significance for multiple comparison, by use of the Bonferroni correction to select the significant coefficients, with the expected fraction of false positives equal to 0.05. Then we calculate a new causality index by summing only the $\{r_{i'}\}$ that pass the FDR test, thus obtaining a filtered linear Granger causality index:

(3)

It is assumed that $\delta_{F}$ measures the causality$y\to x$. By exchanging the roles of the two time series, we may evaluate the causality index$\delta_{F}(x\to y)$.

Kernel Granger Causality (KGC, Marinazzo et al., 2008b) is based on the theory of reproducing kernel Hilbert spaces. Given a kernel function $K$, this methods performs linear Granger causality in the space of the eigenfunctions of K, which are nonlinear in the original variables. An efficient procedure to control the complexity of the model is also proposed, leading to a filtered causality index in the nonlinear case. Thus, kernel algorithms work by embedding data into a Hilbert space and searching for linear relations in that space. In this way, the order of nonlinearity can be easily controlled with the choice of the proper kernel. We have to note at this stage that not all kernels may be used to evaluate KGC. Indeed, if *Y* is statistically independent of *X* and *x*, then $\tilde{\text{x}}$ and should coincide in the limit N→∞. This property of invariance is satisfied only by suitable kernels (Ancona, 2008). In the following we consider two choices for the kernel: the inhomogeneous polynomial (IP) of integer order$p$, which has the form:

$K_{p}\left( X,X^{'} \right)={(1+X^{T}X^{'})}^{p}$,

and the Gaussian kernel:

$K_{\sigma}\left( X,X^{'} \right)=\exp(-\frac{\left( X-X^{'} \right)^{T}(X-X^{'})}{2\sigma^{2}})$.

It is worth to mention that KGC with IP and p=1 is analogous to the standard linear GC, apart from the evaluation of statistical significance, that in KGC is performed on the correlation coefficients.

In the case of M simultaneously recorded time series, we can denote

$X{(c)}_{i}{=(x\left( c \right)_{i},\cdots,x\left( c \right)_{i+m-1})}^{T}$,

for *c=1,….M* and *i=1,…N*. In order to evaluate the causality {x(a)}→{x(b)}, we define, for *i=1,…,N*,

$$Z_{i}=(X(1)_{i}^{T},...,X(a)_{i}^{T},...,X(M)_{i}^{T})^{T},$$

which contains all the input variables, and

$$X_{i}=(X(1)_{i}^{T},...,X(M)_{i}^{T})^{T},$$

which contains all the input variables but those related to {x(a)}. Evaluating the causality a→b for all the pairs, the causality pattern can be fully reconstructed for all the variables.
